# *C. elegans* BLOC-1 Functions in Trafficking to Lysosome-Related Gut Granules

**DOI:** 10.1371/journal.pone.0043043

**Published:** 2012-08-15

**Authors:** Greg J. Hermann, Emily Scavarda, Allison M. Weis, Daniel S. Saxton, Laura L. Thomas, Rebecca Salesky, Hannah Somhegyi, Thomas P. Curtin, Alec Barrett, Olivia K. Foster, Annalise Vine, Katherine Erlich, Elizabeth Kwan, Beverley M. Rabbitts, Kaila Warren

**Affiliations:** 1 Department of Biology, Lewis and Clark College, Portland, Oregon, United States of America; 2 Program in Biochemistry and Molecular Biology, Lewis and Clark College, Portland, Oregon, United States of America; University of North Carolina at Chapel Hill, United States of America

## Abstract

The human disease Hermansky-Pudlak syndrome results from defective biogenesis of lysosome-related organelles (LROs) and can be caused by mutations in subunits of the BLOC-1 complex. Here we show that *C. elegans glo-2* and *snpn-1*, despite relatively low levels of amino acid identity, encode Pallidin and Snapin BLOC-1 subunit homologues, respectively. BLOC-1 subunit interactions involving Pallidin and Snapin were conserved for GLO-2 and SNPN-1. Mutations in *glo-2* and *snpn-1*,or RNAi targeting 5 other BLOC-1 subunit homologues in a genetic background sensitized for *glo-2* function, led to defects in the biogenesis of lysosome-related gut granules. These results indicate that the BLOC-1 complex is conserved in *C. elegans*. To address the function of *C. elegans* BLOC-1, we assessed the intracellular sorting of CDF-2::GFP, LMP-1, and PGP-2 to gut granules. We validated their utility by analyzing their mislocalization in intestinal cells lacking the function of AP-3, which participates in an evolutionarily conserved sorting pathway to LROs. BLOC-1(−) intestinal cells missorted gut granule cargo to the plasma membrane and conventional lysosomes and did not have obviously altered function or morphology of organelles composing the conventional lysosome protein sorting pathway. Double mutant analysis and comparison of AP-3(−) and BLOC-1(−) phenotypes revealed that BLOC-1 has some functions independent of the AP-3 adaptor complex in trafficking to gut granules. We discuss similarities and differences of BLOC-1 activity in the biogenesis of gut granules as compared to mammalian melanosomes, where BLOC-1 has been most extensively studied for its role in sorting to LROs. Our work opens up the opportunity to address the function of this poorly understood complex in cell and organismal physiology using the genetic approaches available in *C. elegans*.

## Introduction

Lysosome-related organelles (LROs) are cell-type specific compartments found in single celled eukaryotes and animals [1], [2]. They share characteristics with conventional degradative lysosomes, such as being acidified and being derived from endocytic trafficking pathways. However, LROs have specialized functions in synthesis, storage, and secretion and they often co-exist with conventional lysosomes [3]. In *C. elegans*, gut granules are intestinal cell specific LROs [4], containing conspicuous autofluorescent and birefringent material [5], [6], which function in zinc storage [7].

Defective formation of LROs underlies the human disease Hermansky-Pudlak syndrome (HPS). The nine genes currently implicated in causing HPS encode subunits of the AP-3, BLOC-1, BLOC-2, or BLOC-3 complexes [8], [9], [10]. Three forms of HPS result from mutations in Dysbindin/HPS-7, BLOS3/HPS-8, and Pallidin/HPS-9, which are BLOC-1 subunits. Loss of BLOC-1 function leads to alterations in the biogenesis of mammalian LROs including melanosomes and platelet dense granules [9]. BLOC-1 is localized to early endosomes and promotes the trafficking of specific proteins from early endosomes to LROs and synaptic vesicles [11], [12], [13], [14]. How BLOC-1 mediates protein sorting to these compartments however, is currently unknown.

Mammalian BLOC-1 is composed of at least 8 subunits, some of which appear to be broadly conserved in eukaryotes, leading to the suggestion that BLOC-1 is an ancient regulator of intracellular trafficking [15], [16]. To date, the best experimental support of this idea comes from studies in *D. melanogaster* showing conserved interactions of BLOC-1 subunits and altered lysosome-related pigment granules when the function of the BLOC-1 encoding subunit *blos1* is disrupted [17]. However, it is unclear from these studies whether protein trafficking to pigment granules is altered in BLOC-1(−), leaving open the question of how analogously BLOC-1 functions in lineages distinct from mammals.

In this report, we provide evidence that BLOC-1 activity is conserved in *C. elegans*: (1) physical interactions between *C. elegans* BLOC-1 subunit homologues are similar to what has been observed in other systems; (2) disrupting the activity of BLOC-1 results in altered trafficking to, and formation of, gut granules; and (3) phenotypic and genetic studies point to BLOC-1 having functions independent of AP-3.

## Materials and Methods

### Nematode Strains and Culture

All *C. elegans* strains were grown at 22°C (unless specifically noted) on NGM media seeded with OP50 *E. coli* as described [18]. The following alleles were used*: aex-4(ok614)*, *aex-4(sa22)*, *apt-6(ok429)*, *apt-7(tm920)*, *dpy-5(e61)*, *dpy-13(e184)*, *glo-1(zu437)*, *glo-2(tm592)*, *glo-2(zu455)*, *hDf6*, *let-502(h392)*, *rrf-3(pk1426), snpn-1(tm1892)*, *syn-13(tm1198)*, *syn-13(tm1764)*, *syn-13(tm2037)*, *unc-5(e53)*, *unc-13(e450), unc-57(e406)*. N2 was used as the wild-type strain. Animals containing the following transgenes were used: *amIs2[cdf-2::gfp; unc-119(+)]I*, *amIs4[cdf-2::gfp; unc-119(+)]*, *enEx413[pced-1::cnts-1::gfp; unc-76(+)]*, *kxEx9[gfp::glo-1; Rol-6^D^]*, *kxEx98[pgp-2::gfp; Rol-6^D^]*, *kxEx141[cpr-6::mCherry; Rol-6^D^]*, *kxEx148[F11E6.1::mCherry; Rol-6D]*, *kxEx209[glo-2p::glo-2.a::gfp; Rol-6^D^]*, *pwIs50[lmp-1::gfp; unc-119(+)]*, *pwIs72[vha-6p::gfp::rab-5; unc-119(+)]*, *pwIs87[vha-6p::rme-1::gfp; unc-119(+)]*, *pwIs170[vha-6p::gfp::rab-7; unc-119(+)]*. References for alleles and transgenes used can be found at Wormbase (www.wormbase.org).

### Yeast 2-hybrid Analysis

The *S. cerevisiae* PJ69-4A strain was used for all 2-hyrbrid assays [19]. Bait and prey constructs were present within GAL4 based plasmids. To generate the pGBKT7g-GLO-2.a and pGBKT7g-SNPN-1 bait plasmids, total RNA was isolated from N2 using Trizol/chloroform extraction, cDNA was generated with Superscript III (Invitrogen, Grand Island, NY, USA) and a d(T)20 primer, and cDNAs encoding each protein were amplified using Phusion DNA polymerase (NEB, Ipswich, MA, USA) with primers that contained attB.1 and attB.2 sites. These PCR products were cloned into a pDONR/Zeo Gateway entry vector with a Gateway BP reaction (Invitrogen). The coding sequence of each gene was sequence verified. Using pDONR201 Gateway entry vectors containing *C. elegans blos-1* and *dsbn-1* cDNAs from Open Biosystems [20] (Lafayette, CO, USA), as well as pDONR/Zeo-GLO-2.a and pDONR/Zeo-SNPN-1 plasmids, we performed Gateway LR reactions with the destination vector pGADT7g [21] to generate the prey plasmids. All bait and prey plasmids were sequence verified prior to use in 2-hybrid assays. The BLOS-4 prey plasmid was identified in a screen for 2-hybrid interactors with the pGBKT7g-GLO-2.a bait plasmid. PJ69-4A containing pGBKT7g-GLO-2.a was transformed with the Caldwell *C. elegans* cDNA library (Addgene, Cambridge, MA, USA) [22] contained within the pACT2.2 prey plasmid using the Yeastmaker transformation system (Clontech, Mountain View, CA, USA). Approximately 200,000 clones were screened on adenine dropout media. 108 clones were isolated and after being recovered from yeast and retested one clone containing the entire *blos-4* cDNA showed a positive interaction with pGBKT7g-GLO-2.a. For the growth assay, overnight cultures of strains containing bait and prey plasmids were grown to saturation in media lacking tryptophan and leucine to select for the vectors. The cell cultures were diluted to an OD_600_ of 0.5 and 4 µl of a 1∶4 dilution was spotted onto SD media lacking tryptophan, leucine, histidine, and adenine (Clontech), where adenine was added back at to the media at 0.02 mg/ml, to select for the plasmids and assess expression of the HIS3 reporter. Empty bait and prey plasmids were pGBKT7g and pGADT7g vectors, respectively. However, the empty pACT2.2 prey plasmid showed identical results (not shown). The cells were incubated for 4 days at 30°C and digitally imaged using a SnapScan 1212 scanner and Scanwise software 2.1.

### Genetic Manipulations

*glo-2(zu455)* was backcrossed to wild type 6 times and *glo-2(tm592)* was backcrossed to wild type 4 times prior to the analyses described here. The breakpoints of the *tm592* deletion were verified by DNA sequencing. A three factor genetic cross placed *glo-2(zu455)* between *dpy-5* and *unc-57*: *dpy-5* (24 recombinants) *glo-2* (11 recombinants) *unc-57*. A three factor genetic cross placed *glo-2(zu455)* between *unc-13* and *let-502*: *unc-13* (25 recombinants) *glo-2* (1 recombinant) *let-502*. A four factor genetic cross positioned *glo-2(zu455)* between CE1-167 (F28B3.4) a SNP in CB4856 and *unc-57*: *dpy-5* (17 recombinants) CE1-167 (3 recombinants) *glo-2 unc-57*. Deficiency mapping showed that *hDf6* removed *glo-2(*−*)*. For studies using *hDf6*, linked *dpy-5(e61*) and *unc-13(e450)* alleles that did not alter the number of autofluorescent gut granules were sometimes present. *glo-2(zu455)* was not *smg* suppressed as *smg-3(ma117); glo-2(zu455)* and *smg-4(ma116); glo-2(zu455)* strains were Glo. To identify *glo-2(*−*)* in crosses analyzing recessivity, maternal, and zygotic effects, a linked *dpy-5(*−*)* marker that did not alter gut granule number was used.

*snpn-1(tm1892)* was backcrossed to wild type 4 times prior to our analyses. The Glo phenotype exhibited by the *snpn-1(tm1892)* strain mapped to the location of *snpn-1(+)* in a three factor cross: *dpy-13* (38 recombinants) *glo* (6 recombinants) *unc-5*. The presence of the *snpn-1(tm1892)* deletion was verified by PCR. Transgenic rescue was used to show that *snpn-1(tm1892)* caused the Glo phenotype. Hot Start Taq (Qiagen, Germantown, MD, USA) was used to amplify *snpn-1(+)* and 600 bp (P804–P805) of flanking sequences from N2 genomic DNA. This fragment was injected into *snpn-1(tm1892)* at 4 ng/µl with the marker pRF4[Rol-6^D^] at 100 ng/µl to generate 6 independent transgenic lines [23], all of which were rescued for embryonic and adult Glo phenotypes. One line *snpn-1(tm1892); kxEx241[snpn-1(+); Rol-6^D^]* was found to have normal trafficking of PGP-2 to gut granules as well. Standard genetic approaches showed that *snpn-1(tm1892)* exhibited a recessive, maternally expressed Glo phenotype.

Double mutants of *glo-2(zu455)* and other *glo* genes were generated by mating a *glo* mutant with a tightly linked recessive visible marker with males homozygous for the other *glo* gene. Resulting progeny were self-crossed and adults exhibiting a Glo phenotype that did not display the visible phenotype were cloned out, thus homozygosing one of the *glo* mutants. Those animals that displayed the visible marker were isolated in the next generation, thus homozygosing the second *glo* gene. In all cases, multiple independent lines were isolated and scored.

RNAi was carried out by feeding [24], with bacterial clones derived from Source BioScience (Nottingham, UK) or Open Biosystems *C. elegans* RNAi libraries.

### Molecular Identification and Characterization of *glo-2*

The fosmid WRM0623aC05 was injected at 5 ng/µl with the marker pRF4[Rol-6^D^] at 100 ng/µl into *glo-2(*−*)* strains and rescued the adult and embryonic Glo phenotypes of *glo-2(zu455)* in 3/3 lines and *glo-2(tm592)* in 2/2 lines. To map the rescuing activity, a series of overlapping PCR products generated with LongAmp Taq (NEB) were amplified from WRM0623aC05 or N2 genomic DNA and injected with pRF4[Rol-6^D^] at 100 ng/µl into *glo-2(zu455)*. A 17 kb (P688–P683) fragment that encompassed the first three genes of operon CEOP1204 was injected at 2.5 ng/µl and rescued the Glo phenotypes of *glo-2(zu455)* in 2/2 lines. A 14 kb (P687-P683) fragment that encompassed F57C9.4 and F57C9.3 was injected at 2.5 ng/µl and rescued the Glo phenotypes of *glo-2(zu455)* in 2/2 lines. A 7.5 kb (P687-P649) fragment that encompassed F57C9.4 and the first two exons of F57C9.3 was injected at 2.5 ng/µl and did not rescued the Glo phenotypes of *glo-2(zu455)* in 2/2 lines. A 10 kb (P684–P683) fragment that encompassed F57C9.3 was injected at 2.5 ng/µl and rescued the Glo phenotypes of *glo-2(zu455)* in 4/4 lines. A 7 kb (P700–P701) fragment that spanned from the 3′ end of the F57C9.4 coding sequence through the first 3 exons of F57C9.3 was injected at 2.5 ng/µl and rescued the Glo phenotypes of *glo-2(zu455)* in 2/2 lines and *glo-2(tm592)* in 2/2 lines. *clec-90(tm3402)*, a deletion of 5′ end of *clec-90* (F57C9.2) did not display a Glo phenotype.

To determine the gene structure of F57C9.3 we isolated total RNA from N2 using Trizol/chloroform extraction and generated cDNA using Superscript III and a d(T)20 primer. We amplified the predicted (Wormbase WS210) F57C9.3 cDNA (exons 1–5) and sequenced the resulting product to find that it lacked predicted exons 3 and 4. Using RT-PCR we found that F57C9.3 was part of a polycistronic message with the downstream gene *clec-90*. Using an SL2 primer, we found that *clec-90* was trans-spliced. Similarly, RT-PCR and sequence analyses indicated that F57C9.3 was trans-spliced with SL1 and SL2 at its 5′ end. Using 3′RACE (Invitrogen) we found that F57C9.3 had alternately spliced transcripts that differed by the presence of exon 4. The form containing exons 1–4 is termed *glo-2.a* and the form containing exons 1–3 is termed *glo-2.b*. Recently, an EST corresponding to the *glo-2.a* transcript has been sequenced, confirming our gene structure (Genbank FN871861).

The sequence of the *glo-2(zu455)* mutation was identified by using Phusion DNA polymerase to PCR amplify all of the coding and flanking intronic sequences of *glo-2.a*. As *zu455* disrupted the splice site immediately 5′ to exon 4, we generated cDNA from *glo-2(zu455)* and used PCR to analyze its effects on *glo-2* splicing. We found that alternative splicing led to exon 3 of *glo-2* being spliced in frame with exon 2 of *clec-90*. To assess the activity of this transcript and whether the activity of *clec-90* might contribute to gut granule formation, we amplified the 824 bp regulatory region immediately upstream of *glo-2* (P695-P748) with Phusion polymerase and used PCR fusion (P699-P774) to add it to the 5′ end of the *glo-2* exon 1,2,3,*clec-90* exon 2,3,4 cDNA (P752-P739) [25]. The 3′UTR of *clec-90* was amplified (P733-P726) and fused to the 3′ end of the transgene (P700-P775). The *glo-2-clec-90* fusion was injected into wild type at 5 ng/µl with the marker pRF4[Rol-6^D^] at 100 ng/µl and did not lead to dominant Glo phenotypes (4/4 lines). To assess the fusion proteins localization and rescuing activity, the *glo-2* regulatory sequences (P695-P748) and *gfp* (P664-P267from pPD95.75) were amplified and fused with P699-P751. The *glo-2* exon 1,2,3,*clec-90* exon 2,3,4 cDNA (P752-P739) was fused with the *glo-2p::gfp* using P699-P774. The 3′UTR of *clec-90* (P733-P726) was added to the construct by fusion with P700-P775. The *glo-2p::gfp::glo-2-clec-90* fusion was injected into *glo-2(zu455)* at 5 ng/µl with the marker pRF4[Rol-6^D^] at 100 ng/µl and resulted in partial rescue of the embryonic Glo phenotype (5/6 lines). The fusion protein was not obviously associated with membranes in intestinal cells. To assess the rescuing activity of *clec-90*, the *glo-2* regulatory sequences (P699-P755) were fused with *clec-90* amplified from N2 genomic DNA (P756-P725) using P700-P726. The *glo-2p::clec-90* construct was injected at 5 ng/µl with the marker pRF4[Rol-6^D^] at 100 ng/µl and did not rescue the Glo phenotype (5/5 lines) of *glo-2(zu455)*. Our analyses of cDNA from *glo-2(zu455)* identified a second *glo-2* transcript whereby a cryptic splice site 65 bp 5′ of normal splice site altered by *zu455* was used. This is predicted to result in a GLO-2 protein with 93 amino acids added to it C-terminus. RT-PCR analyses indicated that *glo-2.b* is expressed in *glo-2(zu455)*.

To assess the rescuing activity of *glo-2.a* and *glo-2.b*: (1) the 824 bp regulatory sequence 5′ to the start of *glo-2* was amplified (P699-P749) and fused to a *glo-2.a* cDNA (P719-P736) with P700-P735, and (2) the regulatory sequences 5′ to the start of *glo-2* were amplified (P664-P749) and fused to the *glo-2.b* cDNA (P719-P721) with P699-P722 followed by amplification of the 3′UTR from N2 genomic DNA (P694-P704) and fusion to the *glo-2p::glo-2.b* construct with P700-P773. These constructs were individually injected into *glo-2(zu455)* at 5 ng/µl with the marker pRF4[Rol-6^D^] at 100 ng/µl. Both *glo-2.a* (4/4 lines) and *glo-2.b* (3/3 lines) constructs rescued the Glo phenotypes of *glo-2(zu455)*.

Multiple secondary structure prediction algorithms including SSpro [26], PHD [27], PSIPRED [28], and JPRED3 [29] indicated that GLO-2 is largely alpha-helical. GLO-2 heptad repeats were not detected used COILS [30] or Marcoil [31], however they were supported by analysis with SOSUIcoil [32].

### GLO-2 Reporters and Antibodies

Carboxy-terminal GFP fusions of GLO-2.a and GLO-2.b were generated by amplifying the 824 bp regulatory sequence 5′ of *glo-2* (P664-P749) and fusing it to the *glo-2.a* cDNA (P719-P735) or *glo-2.b* cDNA (P719-P721) with P699-P753. *gfp* and *unc-54* 3′UTR were amplified from pPD95.75 (P266-P742) and fused with *glo-2p::glo-2.a* using P700-P267. *gfp* and *unc-54* 3′UTR were amplified from pPD95.75 (P267-P783) and fused with *glo-2p::glo-2.b* using P700-P266. These were individually injected into *glo-2(zu455)* at 5 ng/µl with the marker pRF4[Rol-6^D^] at 100 ng/µl. Both *glo-2.a::gfp* (5/5 lines) and *glo-2.b::gfp* (4/6 lines) constructs rescued the Glo phenotypes of *glo-2(zu455)*. One of the resulting lines, *glo-2(zu455); kxEx209[glo-2p::glo-2.a::gfp; Rol-6^D^]* was imaged in this work. An amino-terminal GFP fusion of GLO-2.a was generated by amplifying the 5′ regulatory sequence upstream of *glo-2* (P664-P748) and fusing it to *gfp* amplified from pPD95.75 (P664-P267) with P699-P751. The *glo-2.a* cDNA (P752-P736) was amplified and fused to *glo-2p::gfp* with P700-P735. Injection of the *glo-2p::gfp::glo-2.a* construct into *glo-2(zu455)* at 5 ng/µl with the marker pRF4[Rol-6^D^] at 100 ng/µl resulting in rescue of the Glo phenotype (6/6 lines).

Rabbit affinity purified antibodies were generated against two peptides (1-MSNTEHNVESKNVTDTLDEI20-cys and cys-78KKTSQLELSDTNIEDG93) present in both GLO-2.a and GLO-2.b 2 by Bethyl Laboratories (Montgomery, TX, USA). Only the amino-terminal anti-peptide antibody elicited a signal in immunofluorescence experiments.

### Microscopy

Microscopic analysis was carried out with a Zeiss AxioImager.M2 microscope configured for DIC, polarization, and fluorescence imaging (Thornwood, NY, USA). A Zeiss AxioCam MRm digital camera controlled with AxioVision 4.8 software was used for image capture. In most cases, Z-series were captured and individual optical sections or maximum intensity projections are presented. In all cases, identical imaging and presentation procedures were used for wild-type and mutant strains. Pretzel stage embryos were imaged following the induction of hypoxia by mounting in H_2_O with an excess of living OP50 *E. coli*. Birefringent material present within embryonic intestinal cells was visualized with polarization optics, captured as a Z-stack encompassing the intestine, and presented as a maximum intensity projection. The numbers of birefringent granules were approximated and the localization was determined relative to the position of the lumen seen by DIC microscopy. Embryos were stained with the vital stains LysoSensor Green (Invitrogen) and Nile Red (Sigma, St. Louis, MI, USA) as described [4], [33], and visualized with Zeiss 38 Ex: BP470/40 Em: BP525/50 and Zeiss 45 Ex: BP560/40 Em: BP630/75 filters, respectively. GFP and mCherry protein fusions were detected with these same filters in living embryos. In cases where both GFP and mCherry fusions were present within the same embryo, the mCherry marker was analyzed first, without exciting GFP as it can photactivate to become a red fluorescent protein in hypoxic conditions [34]. Embryos were fixed and stained with GLO-2 (this work), LMP-1 [35], PGP-2 [36], RAB-5 [37], RAB-7 [38], and VHA-17 [39] antibodies as described [40]. We confirmed the specificity of LMP-1 staining by seeing a complete loss of intestinal signals in *lmp-1(nr2045)* embryos. Similarly, we confirmed that RAB-7 staining was lacking in *rab-7(ok511)* embryos. In experiments of fixed CDF-2::GFP, CNTS-1::GFP, GFP::RAB-5, GFP::RAB-7, LMP-1::GFP, and RME-1::GFP expressing embryos, the endogenous fluorescence of GFP was used to visualize the tagged proteins. mCherry fusions were always visualized in living embryos. For analyses of organelle number and morphology, typically at least 40 similarly staged embryos of each mutant genotype were compared to wild-type. To quantify the number of LMP-1::GFP compartments in int2 and int3 intestinal cells [40], Z-stacks containing the entire intestine of fixed 1.5-fold stage embryos were analyzed. Random optical sections of 1.5-fold stage embryos were analyzed to determine the fraction of LMP-1::GFP that co-localized with endolysosomal organelle markers. Larvae and adults were immobilized prior to imaging by mounting in 10 mM levamisole (Sigma) or 0.1 µm polystyrene nanobeads (Polysciences, Warrington, PA, USA). Autofluorescent material within gut cells was visualized with a Zeiss 38 filter except in cases where vital dyes or GFP fusions were also present, when a Zeiss 49 Ex: G365 Em: 445/50 filter was used. The number of autofluorescent granules in each individual was approximated. The vital dyes, LysoTracker Red (Invitrogen), TRITC-Dextran (Invitrogen), Nile Red (Sigma) were used to stain adults as described [4], and visualized with a Zeiss 45 filter.

## Results

### Molecular and Genetic Characterization of *glo-2*

**Figure 1:**
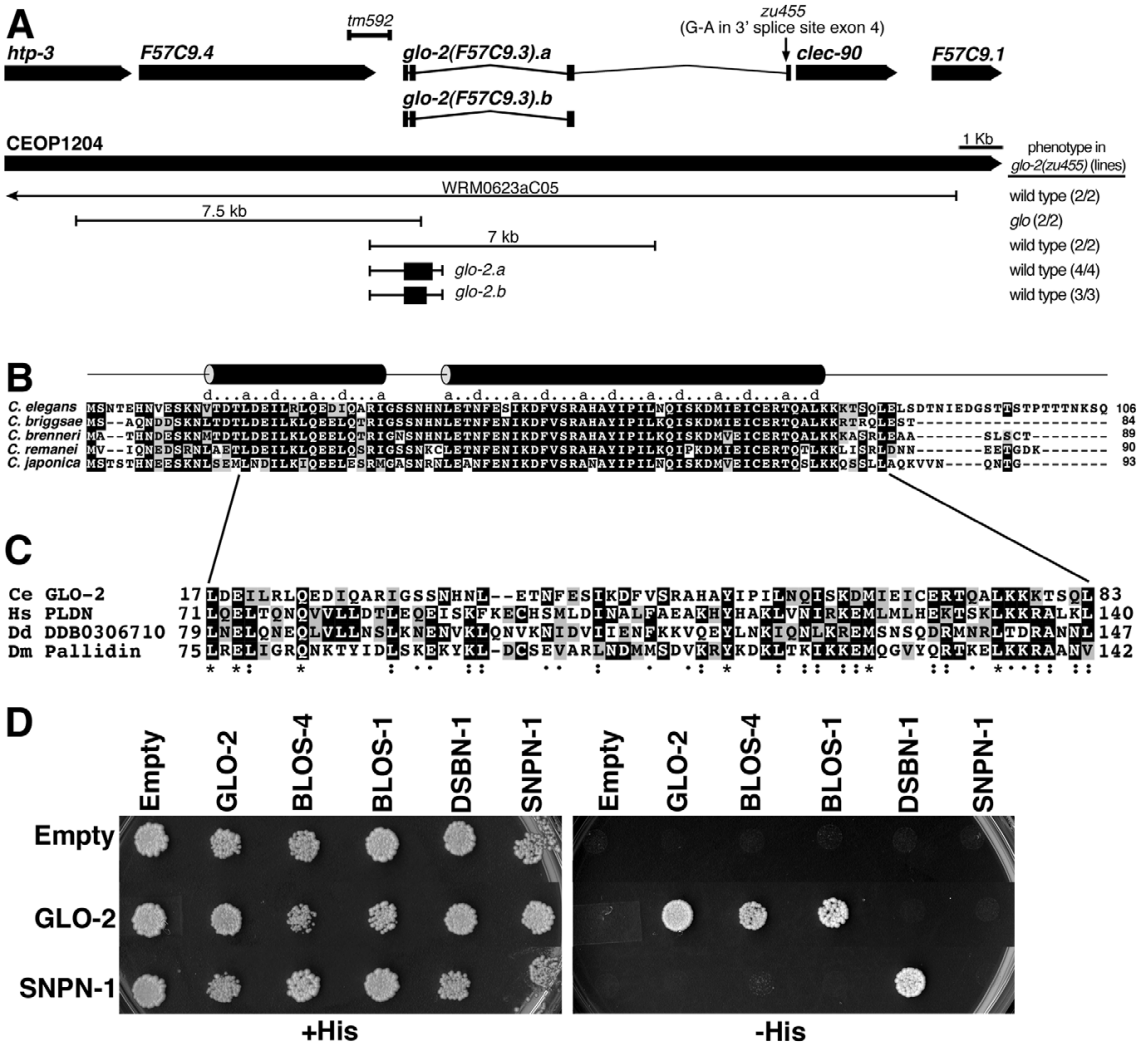
GLO-2 is homologous to Pallidin. (A) *glo-2* is part of operon CEOP1204. The *glo-2* gene structure, sites of *glo-2* mutations, and regions of DNA used in rescue experiments are shown. (B) The predicted sequence of GLO-2.a is aligned to predicted GLO-2 sequences from other nematodes. The cylinder above the sequences denote regions predicted to be alpha helical and a and d mark sites of hydrophobic residues in predicted heptad repeats. (C) Alignment of *C. elegans* GLO-2 with Pallidin from human (Hs, NP_036520), *Dictyostelium* (Dd, XP_635482), and *D. melanogaster* (Dm, NP_648494). (D) Mapping interactions of *C. elegans* BLOC-1 homologues using the yeast 2-hybrid system. Yeast cells were cotransformed with plasmids encoding GAL4 DNA binding domain (left row) and GAL4 activation domain fusions (top row). Cotransformed cells were spotted on the indicated medias. Growth on –His media is indicative of a protein-protein interaction.

*glo-2* was identified in a genetic screen for mutants that display a Glo phenotype, which is characterized by the loss and/or mislocalization of the birefringent and autofluorescent material normally contained within gut granules [4]. We used standard approaches to molecularly identify *glo-2* as F57C9.3. *glo-2(zu455)* mapped to the 200 kb *let-502* to CE1-167 interval of LG I. We analyzed deletion alleles in this region generated by gene knockout consortia for effects on gut granule biogenesis and found that the 950 bp *tm592* deletion displayed reduced numbers of birefringent and autofluorescent gut granules (Tables 1 and 2), which was most penetrant at lower temperatures. *tm592* removes the last exon of F57C9.4b/c, the second gene of the five gene operon CEOP1204 [41], as well as a substantial portion of the intergenic region upstream of F57C9.3 (Figure 1A). The introduction of fosmid WRM0623aC05, which contains the wild-type sequence of nearly the entire CEOP1204 operon, restored wild-type numbers of autofluorescent and birefringent gut granules to both *glo-2(zu455)* and *tm592*. A 7 kb wild-type genomic region containing just F57C9.3 and approximately 800 bp of upstream sequences rescued the Glo phenotypes exhibited by both mutants (Figure 1A). We used RT-PCR and 5′/3′ RACE to determine the exon coding structure of *glo-2* and found that alternative splicing produces two transcripts, *glo-2.a* that contains 4 exons and *glo-2.b* containing only the first 3 exons, encoding proteins 106 and 94 amino acids, respectively (Figure 1B). Related nematode species are predicted to encode proteins highly homologous to GLO-2.b, however, they do not appear to have highly conserved sequences derived from exon 4 of *glo-2.a* (Figure 1B). DNA sequencing showed that *glo-2(zu455)* had a mutation in the conserved 3′ acceptor splice site in intron 3, leading to the generation of *glo-2.a* transcripts with alternate 3′ exons (see methods for details). We found that the 5′ end of *glo-2* was trans-spliced to both SL1 and SL2, which is indicative of a gene whose expression is regulated by both an operon and an internal 5′ promoter [42], [43]. Expression of *glo-2.a* or *glo-2.b* under control of the 800 bp immediately upstream of *glo-2* was sufficient to rescue the loss of birefringent and autofluorescent gut granules of *glo-2(zu455)* (Figure 1A). Using RT-PCR, we detected expression of *glo-2.b* in *glo-2(zu455)*, however it was apparently insufficient to promote wild type gut granule biogenesis in this background (see methods for details).

**Table 1:** Birefringent gut granules in embryos.

Genotype	% lackingbirefringence inintestinal cells (% thatalso mislocalizedbirefringent materialinto the intestinallumen)	% with 1–15 birefringentgranules in intestinalcells (% that alsomislocalizedbirefringent materialinto the intestinallumen)	% with 20–50 birefringentgranules in intestinal cells(% that also mislocalizedbirefringent material intothe intestinal lumen)	% with >50birefringentgranules inintestinal cells	*n*
Wild type	0	0	0	100	166
*glo-2(zu455)*	100(33)	0	0	0	174
*glo-2(tm592)*	0	0	0	100	157
*glo-2(tm592)*15°C^1^	0	47	5(5)	52	109
*snpn-1(tm1892)*	100(49)	0	0	0	107
*apt-6(ok429)*	6(2)	27(16)	67(5)	0	109
*apt-7(tm920)*	22(6)	43(22)	35(9)	0	106
*glo-1(zu437)*	100(54)	0	0	0	121
**Genotype of Parent**					
*glo-2(zu455)*/*+* 2	0	0	0	100	85
*glo-2(tm592)*/*+* 2	0	0	0	100	91
*glo-2(zu455)*/*glo-2(tm592)* 3	3(1)	49	31(10)	17	249
**Double mutants**				0	
*glo-2(zu455); apt-7(tm920)*	100(33)	0	0	0	52
*glo-2(zu455); glo-1(zu455)*	100(32)	0	0	0	60
*snpn-1(tm1892); apt-6(ok429)*	100(56)	0	0	0	80
*snpn-1(tm1892); apt-7(tm1892)*	100(40)	0	0	0	52
*snpn-1(tm1892); glo-1(zu437)*	100(59)	0	0	0	53
**BLOC-1 interacting SNAREs**					
*aex-4(ok614)*	0	0	0	100	43
*aex-4(sa22)*	0	0	0	100	48
*syn-13(tm1198)*	0	0	0	100	35
*syn-13(tm1764)*	0	0	0	100	44
*syn-13(tm2037*)	0	0	0	100	36

All strains were grown at 22°C, unless noted. Three-fold and later stage embryos were analyzed using polarization microscopy and scored for the presence and localization of birefringent material in the intestine. *n*  =  number of embryos scored.

1 Embryos were derived from *glo-2(tm592)* adults grown at 15°C. The phenotype of wild type and *glo-2(zu455)* were not altered by growth at 15°C (data not shown).

2 The progeny of *glo-2(*−*)*/+ animals were scored. ¼ of the progeny were expected to be *glo-2(*−*)*/*glo-2(*−*)*.

3 Reciprocal crosses of *glo-2(tm592)* males with *glo-2(zu455)* hermaphrodites and *glo-2(zu455)* males with *glo-2(tm592)* hermaphrodites were performed to generate *glo-2(tm592)*/*glo-2(zu455)* adults. Pretzel-stage progeny of these animals were scored and were predicted to be ½ *glo-2(tm592)*/*glo-2(zu455)*, ¼ *glo-2(tm592)*/*glo-2(tm592)*, and ¼ *glo-2(zu455)*/*glo-2(zu455)*.

**Table 2:** Autofluorescent gut granules in L4/adults.

	% of animals with the specified number of autofluorescent granules in anterior intestinal cells							
Genotype	0	1–20	21–50	51–100	101–150	>150	*n*	
Wild type	0	0	0	0	0	100	76	
*glo-2(zu455)*	0	46	54	0	0	0	63	
*glo-2(tm592)*	0	0	0	52	48	0	85	
*glo-2(zu455)* 1	0	0	0	0	0	100	75	
*glo-2(tm592)* 1	0	0	0	0	0	100	103	
*glo-2(zu455)/+* 2	0	0	0	0	0	100	29	
*glo-2(tm592)/+* 2	0	0	0	0	15	85	46	
*snpn-1(tm1892)*	100	0	0	0	0	0	67	
*apt-6(ok429)*	23	57	20	0	0	0	81	
*apt-7(tm920)*	4	56	40	0	0	0	71	
*glo-1(zu437)*	100	0	0	0	0	0	74	
**Genotype of parent**								
*+/hDf6* 3	0	0	0	0	0	100	123	
*glo-2(zu455)/hDf6* 4	0	61	39	0	0	0	99	
*glo-2(tm592)/hDf6* 4	0	0	0	74	26	0	145	
*glo-2(zu455)/glo-2(tm592)* 5	0	0	43	57	0	0	216	
**Double Mutants**								
*glo-2(zu455); apt-7(tm920)*	100	0	0	0	0	0	38	
*glo-2(zu455); glo-1(zu437)*	100	0	0	0	0	0	53	
*snpn-1(tm1892); apt-6(ok429)*	100	0	0	0	0	0	44	
*snpn-1(tm1892); apt-7(tm920)*	100	0	0	0	0	0	38	
*snpn-1(tm1892); glo-1(zu437)*	100	0	0	0	0	0	44	
**BLOC-1 interacting SNAREs**								
*aex-4(ok614)*	0	0	0	0	0	100	30	
*aex-4(sa55)*	0	0	0	0	0	100	26	
*syn-13(tm1198)*	0	0	0	0	0	100	22	
*syn-13(tm1764)*	0	0	0	0	0	100	24	
*syn-13(tm2037)*	0	0	0	0	0	100	23	

All strains were grown at 22°C. Individual L4 stage larvae/young adults were analyzed using fluorescence microscopy with a fluorescein isothiocyanate (FITC) filter and were scored for the number of autofluorescent organelles within the intestinal cells located between the pharynx and vulva. *n* = number of animals scored.

1 *glo-2(*−*)*/*glo-2(*−*)* progeny of *glo-2(*−*)*/*glo-2(+)* hermaphrodite parents were scored.

2 *glo-2(*−*)*/*glo-2(*−*)* hermaphrodites were mated with *glo-2(+)*/*glo-2(+)* males and the resulting *glo-2(*−*)*/*+* animals were scored.

3 *hDf6*/+ heterozygotes were generated by mating *dpy-5(*−*)*/+ males with *unc-13(*−*) dpy-5(*−*) hDf6; hDp31* hermaphrodites and selecting Dpy non-Unc progeny, which were *dpy-5(*−*)*/*unc-13(*−*) dpy-5(*−*) hDf6*. These were allowed to self-cross. Homozygous *hDf6* is recessive embryonic lethal [44] so 2/3 of the resulting progeny were predicted to be +/*hDf6* and 1/3 were predicted to be +/+.

4 *glo-2(*−*)*/*hDf6* animals were generated by mating *glo-2(*−*) dpy-5(*−*)*/+ + males with *unc-13(*−*) dpy-5(*−*) hDf6; hDp31* hermaphrodites and selecting Dpy non-Unc progeny, which were *glo-2(*−*) dpy-5(*−*)*/*unc-13(*−*) dpy-5(*−*) hDf6*. These were allowed to self-cross. Homozygous *hDf6* is recessive embryonic lethal [44], so 2/3 of the resulting progeny were predicted to be *glo-2(*−*)*/*hDf6* and 1/3 were predicted to be *glo-2(*−*)*/*glo-2(*−*)*. The number of autofluorescent compartments in *glo-2(zu455)*/*hDf6* was not significantly decreased compared to *glo-2(zu455)* χ^2^ P = 0.07. The number of autofluorescent compartments in *glo-2(tm592)*/*hDf6* was decreased compared to *glo-2(tm592)* χ^2^ P = 0.001.

5 Reciprocal crosses of *glo-2(tm592)* males with *glo-2(zu455)* hermaphrodites and *glo-2(zu455)* males with *glo-2(tm592)* hermaphrodites were performed to generate *glo-2(tm592)*/*glo-2(zu455)* adults. The L4 stage progeny of these animals were scored and were predicted to be ½ *glo-2(tm592)*/*glo-2(zu455)*, ¼ *glo-2(tm592)*/*glo-2(tm592)*, and ¼ *glo-2(zu455)*/*glo-2(zu455)*.

**Figure 2:**
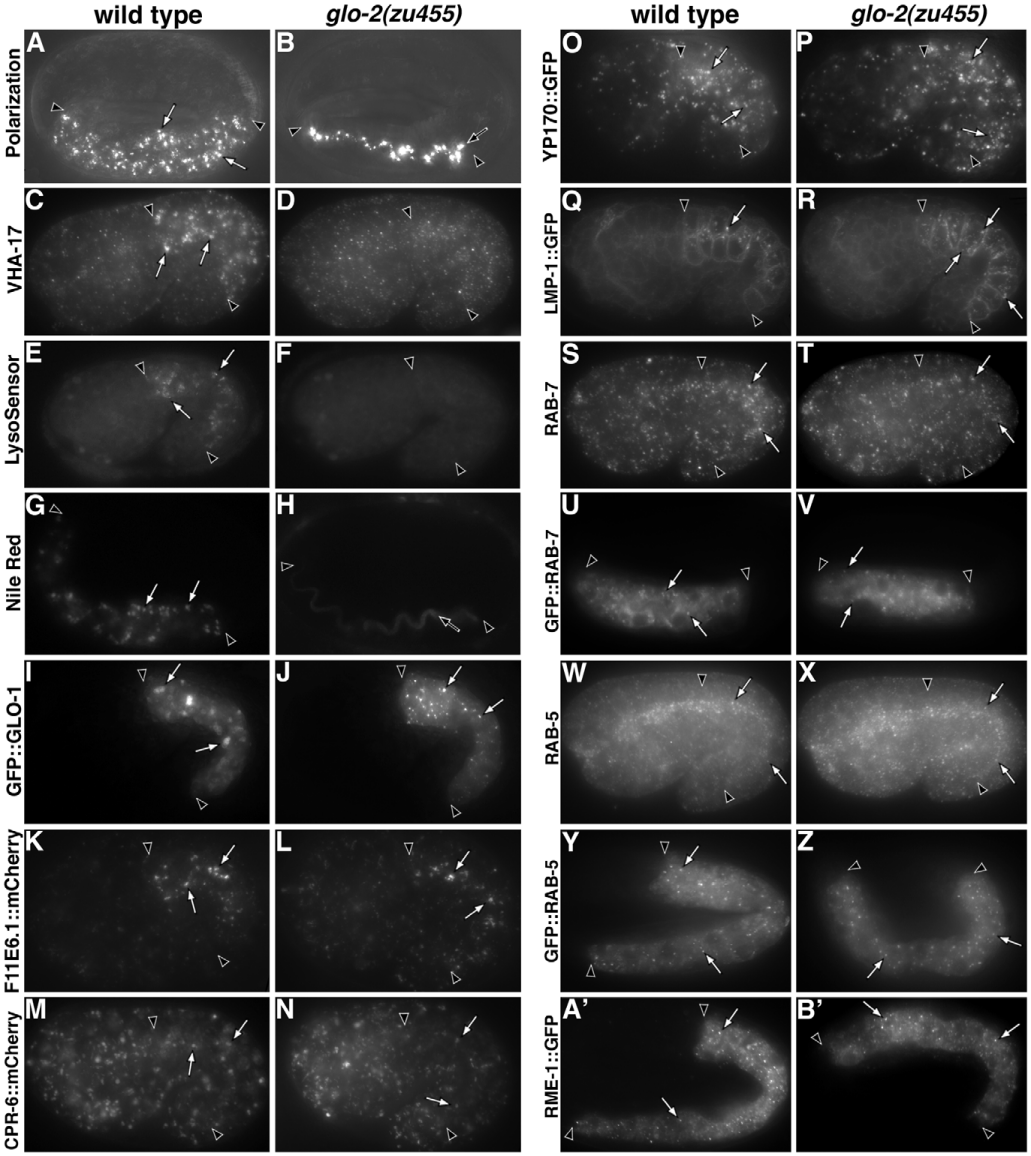
Analysis of gut granules and endolysosomal compartments in *glo-2(zu455)* embryos. (A–B) Birefringent material contained within wild-type gut granules (white arrows) was mislocalized to the intestinal lumen (marked by black arrow) in *glo-2(*−*)* embryos. In wild-type, gut granules (white arrows) were acidified, containing the V-ATPase subunit VHA-17 (C) and were stained by the acidophilic dye LysoSensor Green (E). *glo-2(*−*)* embryos lacked prominent VHA-17 containing organelles (D) and LysoSensor Green stained intestinal organelles (F). (G-H) Nile Red accumulated within wild type-gut granules (white arrows) and was lacking from *glo-2(*−*)* embryos, instead it marked the intestinal lumen (black arrow). (I) GFP::GLO-1 marked the periphery of gut granules in wild type (white arrows). (J) In *glo-2(*−*)*, GFP::GLO-1 marked small puncta morphologically distinct from gut granules (white arrows). (K–N) The numbers and morphology of conventional lysosomes (white arrows) containing the FllE6.1::mCherry and CPR-6::mCherry hydrolases was similar in wild type and *glo-2(*−*)*. (O-P) Yolk platelets marked with YP170::GFP (white arrows) were indistinguishable between wild type and *glo-2(*−*)*. (Q) In wild type, LMP-1::GFP marked organelles (white arrow) located near the apical/lumenal surface of embryonic gut cells. (R) In contrast, *glo-2(*−*)* embryos displayed increased numbers of LMP-1::GFP compartments that were more basally distributed. Q and R show different focal planes of the intestinal primordium to reveal all of the apically localized compartments in wild type and the basally localized organelles on one side of the *glo-2(zu455)* intestine (this region of wild type lacks LMP-1::GFP). The numbers and morphology of late, early, and recycling endosomes (white arrows), labeled by RAB-7 (S–V), RAB-5 (W–Z), and RME-1 (A′–B′) respectively, were not obviously altered from wild type by *glo-2(*−*)*. The intestine is flanked by black arrowheads in all panels. Pretzel stage embryos are shown in A–B, G–H, U–V, and Y–B′ and 1.5-fold stage embryos are shown in C–F, I–T, and W–X. Embryos are approximately 50 µm in length.

**Figure 3:**
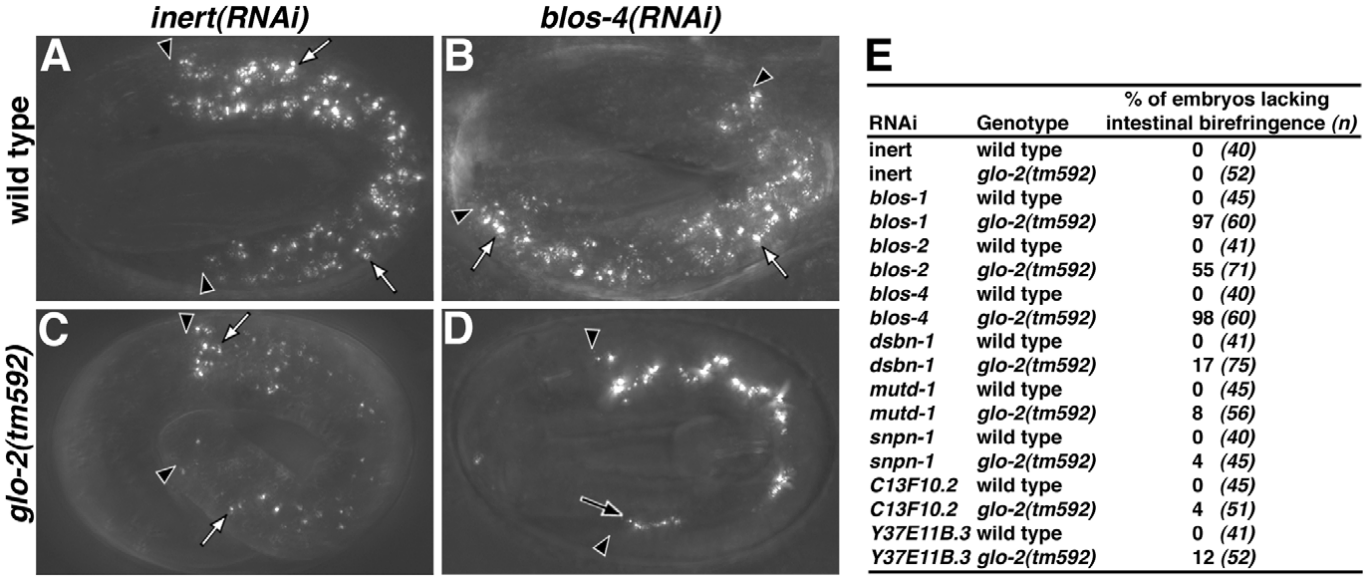
The Glo phenotype of *glo-2(tm592)* is enhanced by RNAi against *C. elegans* BLOC-1 homologues. (A, C) Birefringent gut granules were present in wild-type and *glo-2(tm592)* animals subjected to RNAi targeting F33E2.4 (inert), a gene not required for gut granule formation (white arrows). (B, D) *blos-4(RNAi)* did not alter the appearance of birefringent gut granules in wild type (white arrows), however in *glo-2(tm592)* it led to the loss of birefringent material from intestinal cells and its mislocalization into the intestinal lumen (black arrow). (E) Quantification of intestinal birefringence in >2-fold stage wild-type and *glo-2(tm592)* embryos treated with RNAi targeting BLOC-1 homologues. RNAi targeting all of the genes tested, except *snpn-1* and C13F10.2, led to a significant (Z-test p<0.05) loss of birefringent gut granule material. In A–D, black arrowheads flank the intestine of pretzel stage embryos.

Both *glo-2* alleles, *zu455* and *tm592*, similarly caused recessive, maternal effect Glo phenotypes that could be zygotically rescued (Tables 1 and 2). However, they had distinct effects on the formation of birefringent and autofluorescent gut granules (Tables 1 and 2, data not shown), suggesting that *zu455* and *tm592* differentially compromise *glo-2* activity. *glo-2(zu455)* displayed the strongest defects, exhibiting a complete loss of birefringent material from intestinal cells and its mislocalization into the embryonic intestinal lumen (Figure 2B and Table 1). Some autofluorescent gut granules were present within *glo-2(zu455)* adults (Table 2). *glo-2(tm592)* exhibited a weaker Glo phenotype than *glo-2(zu455)*. Under standard growth conditions *glo-2(tm592)* embryos did not mislocalize birefringent material into the intestinal lumen and always displayed birefringent material in intestinal cells, however with reduced intensity and number when compared to wild type (Figure 3C). Analysis of *glo-2(tm592)* embryos at discreet stages of development showed that alterations in the appearance of birefringent material became less severe as embryogenesis proceeds (data not shown). Multiple markers and proteins associated with gut granules, which were lacking in *glo-2(zu455)* were still present in reduced numbers/intensity in *glo-2(tm592)* (data not shown). When grown at lower temperatures, defects in the formation of birefringent gut granules were accentuated and occasionally *glo-2(tm592)* embryos mislocalized birefringent material into the intestinal lumen (Table 1). The number of autofluorescent gut granules in *glo-2(tm592)* adults was intermediate to wild type and *glo-2(zu455)* (Table 2).

We placed both *glo-2(*−*)* alleles over the *hDf6* deletion [44], which removes *glo-2*, to test whether they exhibited a stronger Glo phenotype, which would be predicted for a partially functional allele. The number of autofluorescent gut granules in *glo-2(zu455)*/*hDf6* adults, while on average was reduced, did not significantly differ from *glo-2(zu455)* (p = 0.07), consistent with *zu455* being a strong loss of function allele (Table 2). In contrast, *glo-2(tm592)*/*hDf6* adults contained less autofluorescent gut granules than *glo-2(tm592)* homozygotes (Table 2) (p = 0.001), indicating that *tm592* has partial function.

As expected for alleles of the same gene, *glo-2(tm592)* and *glo-2(zu455)* did not complement each other for defects in the formation of autofluorescent and birefringent gut granules (Tables 1 and 2). The number of gut granules in *glo-2(tm592)*/*glo-2(zu455)* animals were intermediate to the single mutants (Tables 1 and 2), consistent with *glo-2(tm592)* exhibiting partial activity and *glo-2(zu455)* being a strong loss of function allele. Interestingly, *glo-2(tm592)*/*glo-2(zu455)* animals displayed a stronger defect in the formation of autofluorescent gut granules than *glo-2(tm592)*/*hDf6* (Table 2). If *glo-2(zu455)* was null then both genotypes would have identical phenotypes. Our observations show that *glo-2(zu455)* dominantly inhibits the partial function of *glo-2(tm592)* in gut granule formation, while being recessive to *glo-2(+)* (Table 2). This strongly suggests that *glo-2(zu455)* is not null and while being a strong loss of function allele likely encodes a product with some functional properties.

### *glo-2* Encodes a Pallidin Homologue

GLO-2 encodes two small proteins that differ by 12 amino acids at their carboxy termini (Figure 1A and 1B). Multiple secondary structural prediction algorithms indicate that GLO-2 is largely alpha helical (see methods). Moreover, the predicted alpha helical regions contain heptad repeats suggesting that GLO-2 associates with itself or other proteins by forming coiled coils (Figure 1B). Sequence homology searches showed that GLO-2 is weakly homologous to Pallidin, a small heptad repeat containing protein conserved across metazoans [15], [45]. *C. elegans* GLO-2 shows only 21% identity with human Pallidin, however, the hydrophobic a and d positions of the heptad repeats are well conserved between GLO-2 and Pallidin proteins (Figure 1C). Notably, *C. elegans* GLO-2 isoforms are significantly shorter, at 94/106 aa, than either the *Drosophila* (167 aa) or human (172 aa) Pallidin proteins.

Pallidin functions in the biogenesis of LROs. Mice lacking Pallidin do not properly form melanosomes and platelet dense granules [46], [47] and disruption of human pallidin causes Hermansky-Pudlak Syndrome Type 9, which is characterized by defects in LRO biogenesis and results in severe albinism and excessive bleeding [8]. Thus, despite low levels of amino acid identity, the predicted protein structures and similar roles in LRO biogenesis strongly suggest that GLO-2 is orthologous to Pallidin.

### *C. elegans* BLOC-1 Subunit Homologues Function in Gut Granule Biogenesis

In both mammals and *Drosophila*, Pallidin is a subunit of BLOC-1, a complex composed of at least eight different proteins [48]. Bioinformatics analyses have led to the proposal that the *C. elegans* genome encodes six BLOC-1 subunits [15], [16]. Misprediction of the *glo-2* gene during the original annotation of the *C. elegans* genome prevented the identification of the GLO-2 as a Pallidin homolog in these studies. Despite extensive searches, we and others have been unable to identify a BLOS3 homologue in *C. elegans*. The degree of amino acid identity between *C. elegans* and human or *Drosophila* BLOC-1 subunits range between 14–47% (Table 3). However, despite in some cases low levels of amino acid identity, all of the *C. elegans* genes, like their mammalian and *Drosophila* counterparts, are predicted to encode largely alpha helical, heptad repeat containing, coiled-coil proteins (not shown). With the exception of BLOS-1 and SNPN-1, the *C. elegans* BLOC-1 homologues are shorter than mammalian and *Drosophila* counterparts (Figure S1). Alignments reveal that the *C. elegans* BLOC-1 homologues contain many of the conserved sequence motifs that have been recognized in BLOC-1 subunits [15] (Figure S1).

**Table 3:** *C. elegans* BLOC-1 subunits.

*C. elegans* gene (sequence name)	Human gene (a.a. identity)	*D. melanogaster* gene (a.a. identity)
*blos-1* (T20G5.10)	BLOS1 (47%)	*blos1* (42%)
*blos-2* (Y73B6BL.30)	BLOS2 (29%)	*blos2* (26%)
?	BLOS3	*blos3*
*blos-4* (T24H7.4)	Cappuccino (20%)	*blos4* (18%)
*dsbn-1* (Y106G6A.5)	Dysbindin (21%)	*dysbindin* (18%)
*mutd-1* (C34D4.13)	Muted (14%)	*muted* (17%)
*glo-2* (F57C9.3)	Pallidin (21%)	*pallidin* (17%)
*snpn-1* (C02B10.2)	Snapin (29%)	*snapin* (15%)

The gene name of each *C. elegans* BLOC-1 subunit and its human and *D. melanogaster* orthologues are listed. The amino acid identity of the *C. elegans* protein with each orthologue was derived from pairwise sequence alignments with the full length *C. elegans* protein.

**Figure 4:**
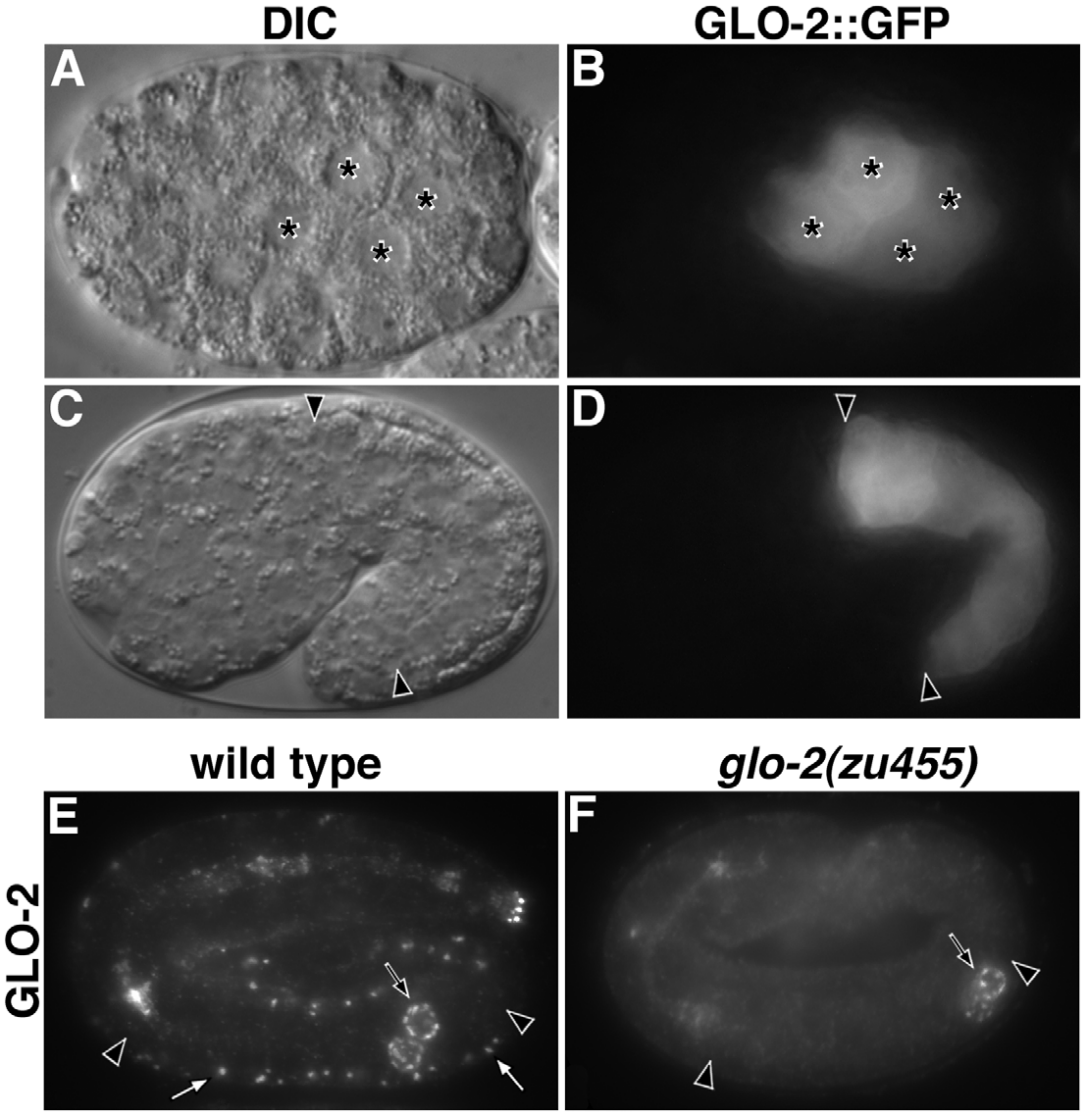
The expression and localization of GLO-2. (A–D) *glo-2::gfp* was expressed under the control of the operon internal promoter immediately upstream of *glo-2*. (A–B) GLO-2::GFP expression was first detected in intestinal precursors (marked with asterisks) at the E^4^ stage of embryogenesis. (C–D) At the 1.5-fold stage, GLO-2::GFP was expressed in intestinal cells (between black arrowheads) and was localized to the cytoplasm. (E–F) Wild-type and *glo-2(zu455)* pretzel-stage embryos stained with anti-GLO-2 antibodies. *glo-2(zu455)* embryos lacked staining of hypodermal organelles by GLO-2 antibodies (white arrows) however they retained staining of P-granules (black arrows). GLO-2 antibodies did not stain prominent organelles within intestinal cells (between black arrowheads). Single focal planes are shown in A–D and maximum intensity projections of 1.25 micron Z-stacks are shown in E–F.

**Figure 5:**
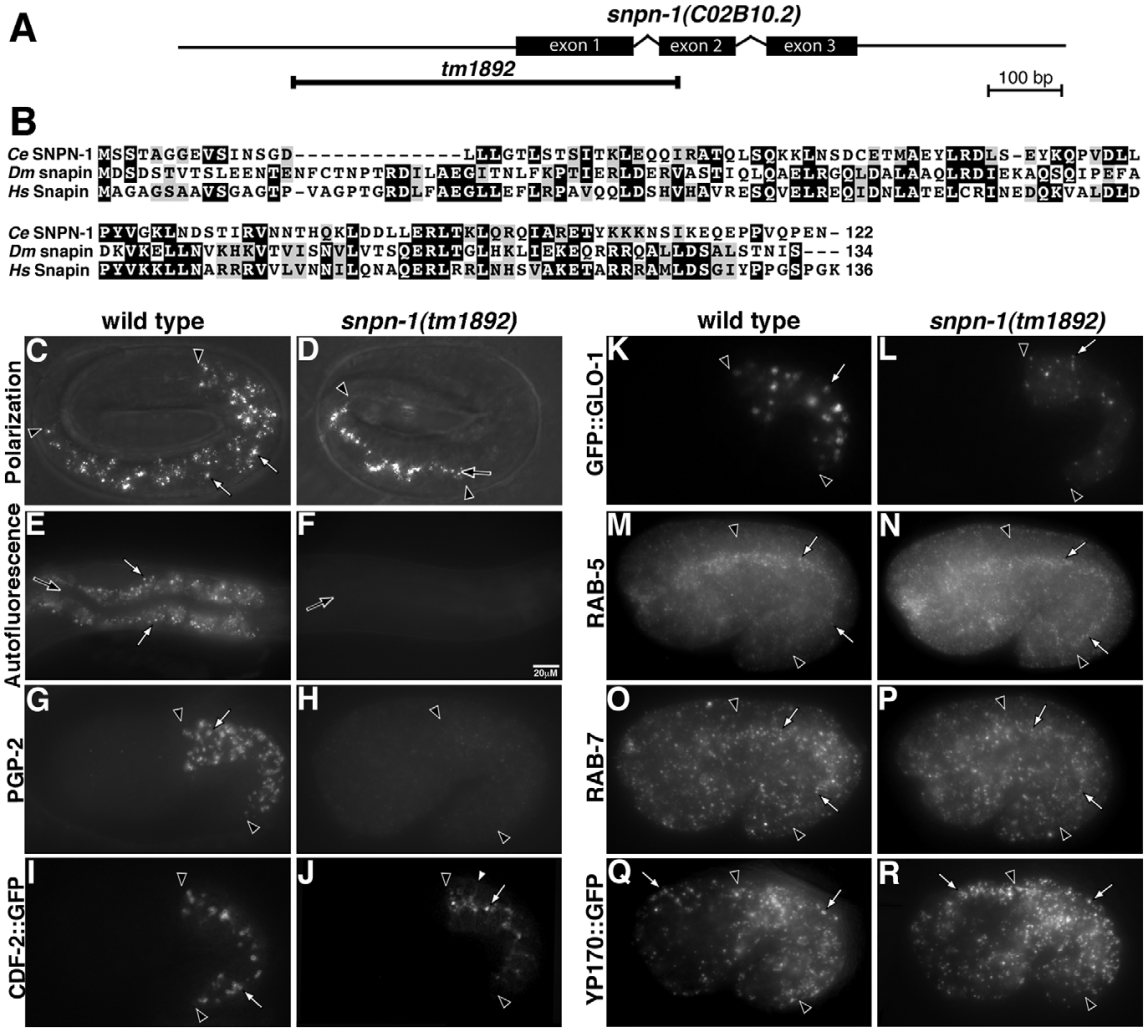
*snpn-1(+)* functions in gut granule biogenesis. (A) The *snpn-1* gene structure and site of the *tm1892* deletion are shown. (B) The predicted sequence of *C. elegans* SNPN-1 is aligned to Snapin from *D. melanogaster* (Dm, NP_722835) and humans (Hs, NP_036569). (C–D) Birefringent material found in wild-type pretzel stage intestinal cells (white arrows) was mislocalized to the intestinal lumen in *snpn-1(tm1892)* (black arrow). (E–F) Autofluorescent gut granules present in wild-type adults (white arrows) were lacking from the intestinal cells of *snpn-1(tm1892)*. (G, I, K) In wild-type embryos, PGP-2, CDF-2::GFP, and GFP::GLO-1 were associated with gut granules (white arrows). In *snpn-1(tm1892)*, PGP-2 was absent from intestinal cells (H), CDF-2::GFP was associated with the plasma membrane (white arrowhead) and organelles located near the apical surface of *snpn-1(tm1892)* intestinal cells (white arrow) (J), and GLO-1::GFP localized to organelles that were significantly smaller than gut granules (white arrow) (L). (M-R) The number and morphology of RAB-5 labeled early endosomes, RAB-7 labeled late endosomes, and YP170::GFP marked yolk platelets (white arrows) were not altered by *snpn-1(tm1892)*. In G–R, 1.5-fold stage embryos are shown. In C–R, the black arrowheads flank the intestine and black arrows mark the intestinal lumen.

To determine whether the other *C. elegans* BLOC-1 homologues function in LRO biogenesis, we used RNAi and genetic mutations to assess their role in the formation of gut granules. In addition, we investigated whether Y37E11B.3 and C13F10.2, genes predicted to encode small largely alpha helical proteins that have recently been suggested to be part of BLOC-1 [16], participate in gut granule biogenesis. In wild-type or *rrf-3(*−*)* RNAi sensitive backgrounds there were no obvious defects in the formation of autofluorescent or birefringent gut granules in BLOC-1 RNAi experiments (Figure 4 B, E, and data not shown). BLOC-1 subunits in *Drosophila* are known to be refractory to RNAi [17]. To genetically sensitize the background, we carried out RNAi against the BLOC-1 homologues in the partial loss of function *glo-2(tm592)* allele. Typically, *glo-2(tm592)* embryos exhibited near normal numbers of gut granules with reduced birefringence (Figure 3C and Table 1). We found that RNAi targeting five of the BLOC-1 homologues significantly enhanced the Glo phenotype of *glo-2(tm592)*, so that many embryos completely lacked birefringent compartments and mislocalized the birefringent material into the intestinal lumen (Figure 3 D–E). Notably, RNAi targeting the proposed BLOC-1 subunit encoding gene Y37E11B.3, which is part of a conserved gene family that has not been functionally characterized in any organism [16], resulted in the loss of birefringent material from gut cells (Figure 3E), suggesting a role in trafficking to LROs. Of the BLOC-1 homologues, only *glo-2* and *snpn-1* are known to have mutant alleles. We found *snpn-1(tm1892)*, which lacks a large region of the *snpn-1* coding sequence and is likely null, results in a complete loss of birefringent and autofluorescent gut granules (Tables 1, 2 and Figure 5C–F). Together these observations show that BLOC-1 homologues in *C. elegans* function in LRO biogenesis.

The mammalian BLOC-1 complex can be dissociated into two stable subcomplexes *in vitro* (Pallidin-Cappucino/BLOS4-BLOS1 and Snapin-Dysbindin-BLOS2). The physical interactions of BLOC-1 subunits within and between these subcomplexes are readily detectable using yeast 2-hybrid assays [17], [49], [50], [51], [52]. We therefore investigated whether *C. elegans* BLOC-1 homologues physically associate similarly to *Drosophila* and human BLOC-1 subunits. Like human Pallidin [49], [51], GLO-2.a interacted with itself (Figure 1D). GLO-2.a also interacted with BLOS-4/Cappuccino and BLOS-1, but not SNPN-1 (Figure 1D), which has been seen for human and *Drosophila* homologues [17], [52]. We did not observe an interaction between GLO-2 and DSBN-1, which has been documented in other systems [17], [52]. However, using our constructs, we detected a conserved interaction between SNPN-1 and DSBN-1 (Figure 1D). Therefore, we conclude that BLOC-1 complex structure, as well as its function, is likely conserved in *C. elegans*.

Based on studies of BLOC-1 interacting proteins it has been proposed that the complex functions in endosomal trafficking by modulating SNARE localization and activity [2], [53]. Mammalian Pallidin physically interacts with Syntaxin 13 and Snapin interacts with SNAP-25 family members [45], [54]. Mutations disrupting the function of intestinally expressed *syn-13* and *aex-4*, the *C. elegans* homologues of Syntaxin 13 and SNAP-23, respectively [55], [56], [57], did not show any obvious defects in gut granule biogenesis (Tables 1 and 2). Thus, these SNAREs play at most a minor role in BLOC-1′s activity promoting *C. elegans* gut granule biogenesis.

### Expression and Localization of GLO-2

To determine how directly GLO-2 might participate in gut granule biogenesis and to analyze how similar its distribution is to mammalian Pallidin, which is found in the cytoplasm and peripherally associated with intracellular membranes [49], [51], we analyzed GLO-2 expression and localization. The expression of genes in a *C. elegans* operon is controlled by the regulatory sequences upstream of the first gene in the operon and in some cases more specifically by internal promoters immediately adjacent to downstream genes in the operon [43]. *glo-2* is the third gene in operon CEOP1204 (Figure 1A) and studies of the first gene in this operon, *htp-3*, have shown that the CEOP1204 operon is expressed in the adult intestine [58] and germline [59]. *glo-2(+)* expressed under the control of regulatory sequence immediately upstream of *glo-2* was sufficient to rescue *glo-2(zu455)*. Thus, the region between F57C9.4 and *glo-2* is likely an internal promoter (Figure 1A). We used this sequence to drive the expression of a *glo-2.a* cDNA with a 3′ *gfp* tag. GLO-2::GFP rescued the loss of birefringent and autofluorescent gut granules in *glo-2(zu455)* embryos and adults, respectively (data not shown), indicating that it was functional and that its temporal and spatial expression could support gut granule biogenesis. Expression of this reporter was first detected during embryonic development in the four intestinal (E^4^ stage) precursors (Figure 4, A–B), which is prior to the appearance of mature gut granules [4], [60], and persisted in intestinal cells up to the pretzel stage of embryogenesis (Figure 4 C–D). At all stages, GLO-2::GFP was cytoplasmically localized in intestinal cells and we were unable to detect any organelle enrichment (Figure 4B, D). To examine endogenously expressed GLO-2, we generated antibodies to the amino terminus of GLO-2. Our anti-GLO-2 antibodies did not mark compartments within intestinal cells (not shown), however they stained prominent organelles within the seam cells of pretzel stage embryos (Figure 4E), which were not present in *glo-2(zu455)* (Figure 4F). While these data point to the intestine as a site of activity for GLO-2, they do not reveal the subcellular compartment(s) upon which it acts to facilitate gut granule biogenesis.

### Endosomal Organelles in *glo-2(*−*)*

**Figure 6:**
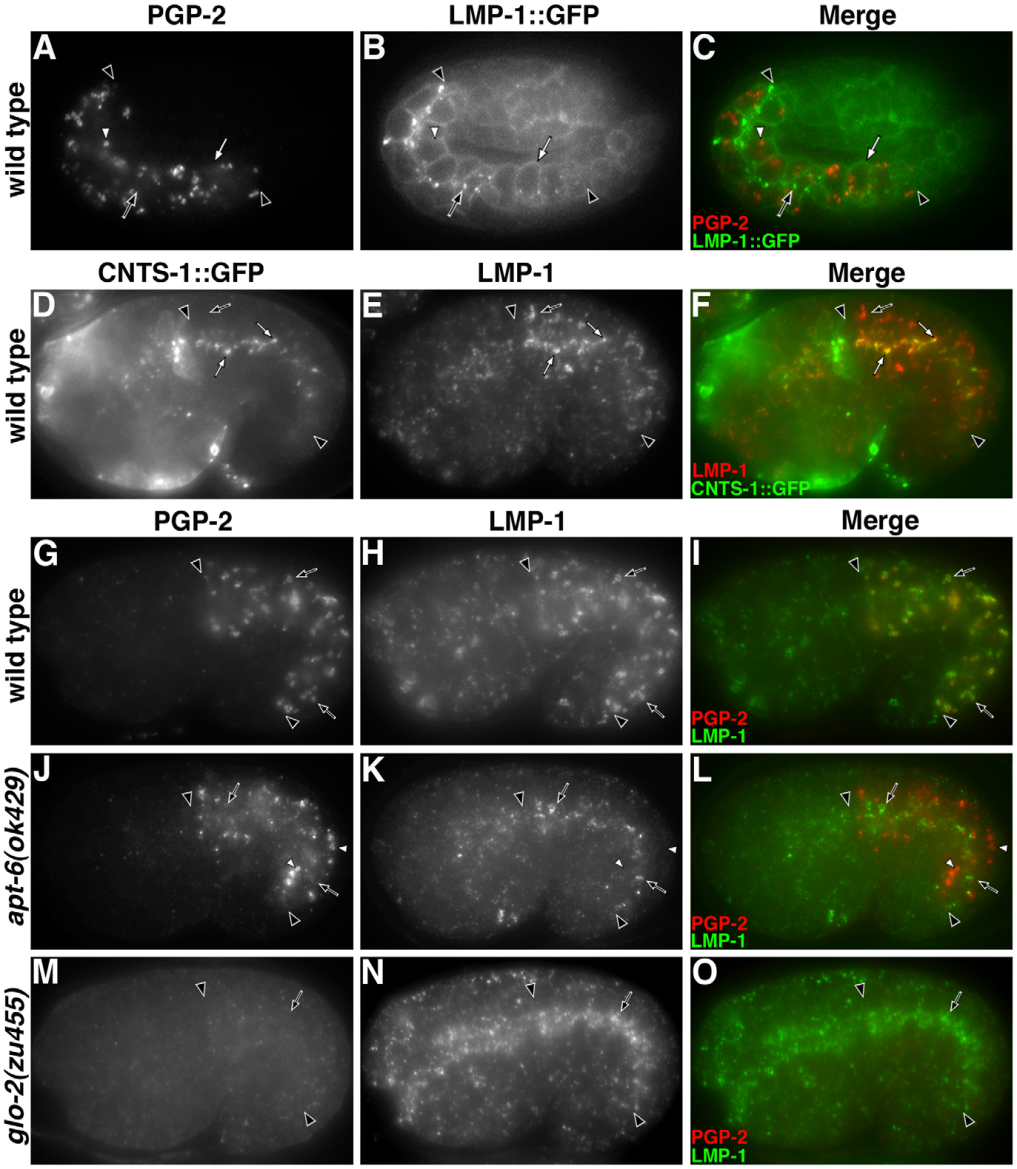
Analysis of LMP-1 trafficking. (A–C) In intestinal cells LMP-1::GFP localized to the basolateral plasma membrane (white arrow) and apically enriched organelles (black arrow) that did not overlap with PGP-2 containing gut granules (white arrowhead). (D–F) Endogenous LMP-1 localized to apical CNTS-1::GFP containing conventional lysosomes (white arrows) and more basally localized compartments (black arrow) lacking CNTS-1::GFP. (G–I) In the basal region of the intestine, LMP-1 localized to PGP-2 containing gut granules (black arrows). (J–L) In a mutant lacking AP-3 function, gut granules containing PGP-2 (white arrowhead) lacked LMP-1, which was restricted to apical compartments (black arrows). (M–O) PGP-2 containing organelles were lacking in *glo-2(zu455)* embryos and LMP-1 was enriched on apically localized organelles (black arrows). In all panels, black arrowheads flank the intestine. In A–C a pretzel stage embryo and in D–O 1.5-fold stage embryos are shown.

To investigate the effects of *glo-2(*−*)* on gut granule biogenesis, we analyzed a number of different gut granule characteristics in *glo-2(zu455)*. Wild-type embryonic gut granules were acidified and stained by LysoSensor dyes, accumulated the lipid dye Nile Red, and contained the V-ATPase subunit VHA-17, the Rab GTPase GLO-1, and the ABC transporter PGP-2 (Figures 2C, E, G, I and 6G). We found that *glo-2(zu455)* embryos lacked PGP-2, LysoSensor Green, acridine orange (data not shown), and Nile Red stained organelles (Figures 2F, 2H, 6M). VHA-17 did not associate with compartments resembling gut granules in *glo-2(zu455)* (Figure 2D). The remaining VHA-17 in the *glo-2(*−*)* intestine, might represent association of VHA-17 with other endocytic compartments or possibly secretory vesicles trafficking VHA-17 to the apical surface of intestinal cells, where it is enriched in late stage embryos [39]. GLO-1, which is orthologous to Rab32/38 in mammals [4], is localized to, and required for, gut granule biogenesis. GFP::GLO-1 was membrane associated in *glo-2(zu455)*, however it localized to organelles that were morphologically distinct from wild-type gut granules (Figure 2I–J). Based upon the subcellular distribution of GLO-1::GFP containing compartments, many are likely distinct from early endosomes and conventional lysosomes, which are enriched near the apical surface of embryonic intestinal cells (Figures 2Q, W). Possibly GLO-1::GFP, which likely cycles on/off membranes in its capacity as a Rab GTPase, becomes enriched on, or mislocalized to, organelles that act as donors in trafficking to gut granules. Together, the dramatic alterations in the gut granule markers we have analyzed indicate that embryonic gut granules are lacking in *glo-2(*−*)*.

**Figure 7:**
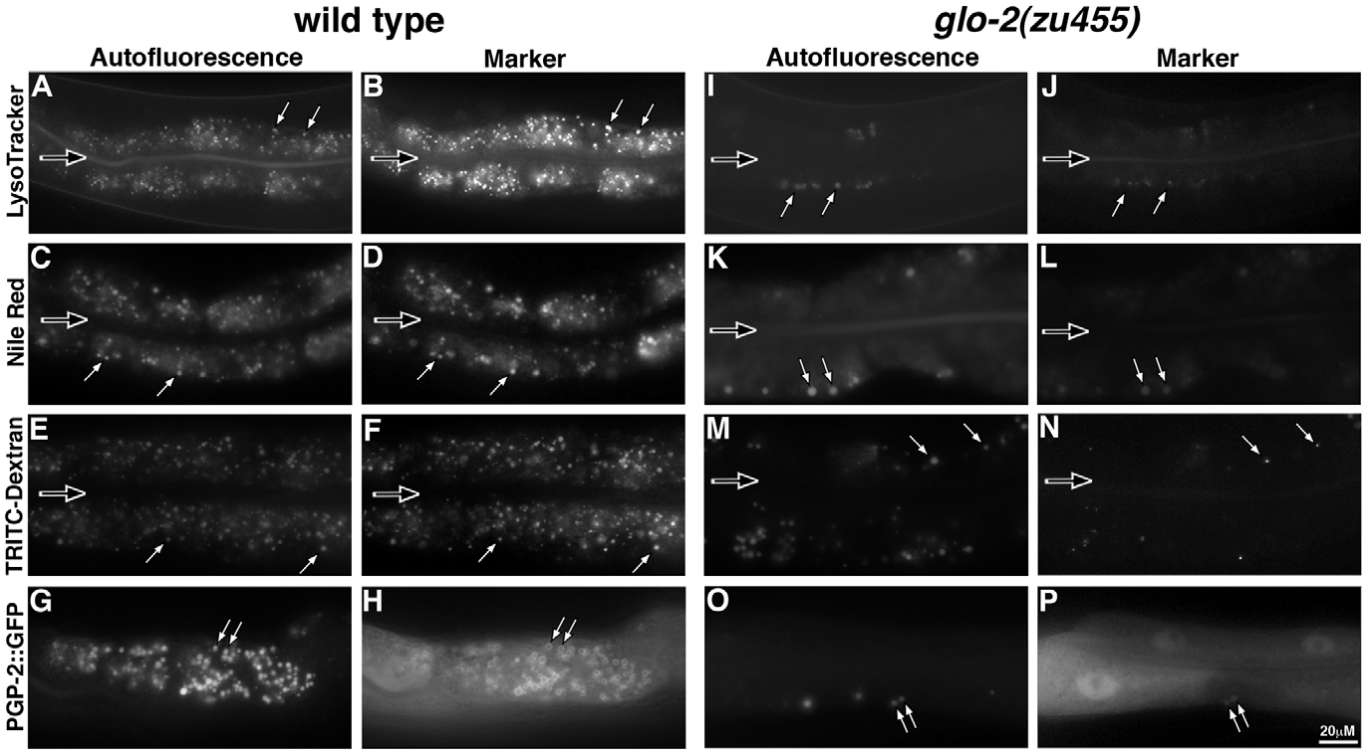
Gut granule formation in adults. In wild-type adults, autofluorescent gut granules accumulated markers of acidification (A–B), hydrophobicity (C–D), terminal endocytic compartments (E–F) and contained PGP-2::GFP (G–H). *glo-2(zu455)* adults had substantially reduced numbers of autofluorescent compartments (I). The majority of these organelles were stained with LysoTracker Red (I-J), Nile Red (K–L) and contained PGP-2::GFP (O–P). (M–N) In contrast, few of the autofluorescent compartments accumulated TRITC-Dextran and those that did localized the marker to a subdomain within the organelle. In all panels, white arrows identify autofluorescent compartments that contained the gut granule marker. The black arrows denote the location of the intestinal lumen.

In wild-type adults, autofluorescent gut granules contained PGP-2::GFP and were acidified, terminal endocytic compartments that accumulated LysoTracker, Nile Red, and TRITC-dextran markers administered by feeding (Figure 7A–H). *glo-2(zu455)* adults contained autofluorescent compartments, albeit many less than are present in wild type (Table 2 and Figure 7I). Many of the autofluorescent organelles in *glo-2(zu455)* exhibited wild type characteristics, containing PGP-2::GFP and being stained by LysoTracker and Nile Red (Figures 7J, L, P). In contrast, few of these organelles accumulated TRITC-dextran (Figure 7N), suggesting that trafficking to and the functional characteristics of adult gut granules were partially disrupted by *glo-2(zu455)*. The GLO-1 Rab GTPase was required for the formation of autofluorescent organelles in *glo-2(zu455)* adults (Table 2), indicating that their formation is likely mediated by normal gut granule biogenesis pathways.

Gut granules are distinct from conventional lysosomes in embryonic intestinal cells [36], [61], [62], thus key aspects of the pathways trafficking cargo to these two organelles must be unique. To investigate whether *glo-2(+)* plays a significant role in the conventional lysosomal trafficking pathway and where *glo-2(+)* functions relative to the divergence of these pathways, we analyzed the formation and morphology of endolysosomal organelles in *glo-2(*−*)*. *glo-2(zu455)* did not dramatically alter the appearance or distribution of RAB-5 containing early endosomes (Figure 2W–Z), RAB-7 containing late endosomes/lysosomes (Figure 2S–V), or RME-1::GFP containing recycling endosomes (Figure 2A′–B′). Conventional lysosomes in intestinal cells contained the soluble glucosylceramidase F11E6.1::mCherry and the cathepsin protease CPR-6::mCherry [33] and their formation and morphology was not obviously altered by *glo-2(zu455)* (Figure 2K–N). In *C. elegans* embryos, alterations in trafficking to lysosomes compromise their degradative properties, which dramatically alters the number and morphology of yolk platelets normally consumed during embryogenesis [63], [64], [65], [66], [67]. We visualized yolk platelets with YP170::GFP [64], and found no obvious differences in yolk platelet number or morphology between *glo-2(zu455)* and wild-type embryos (Figure 2 O–P).

**Figure 8:**
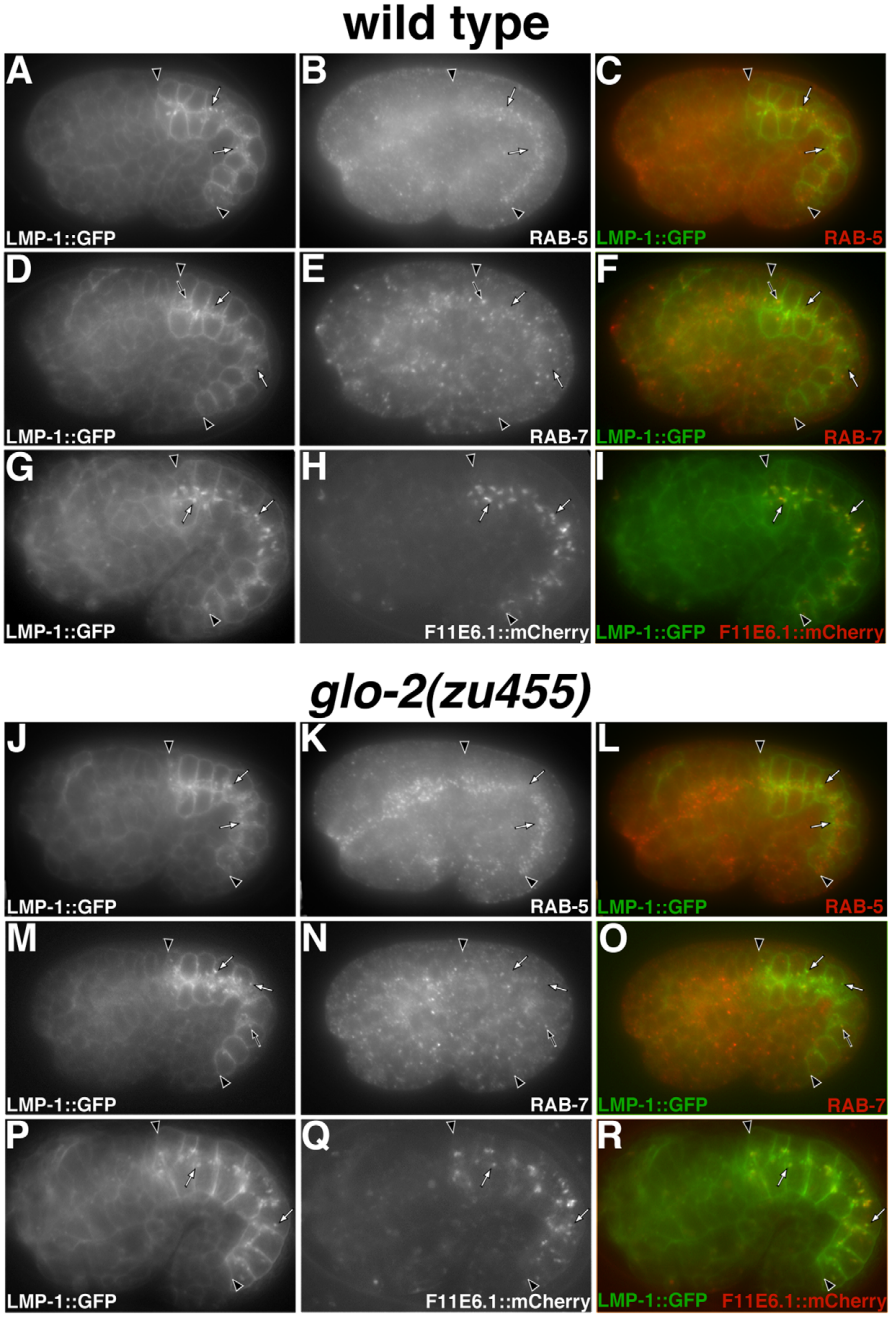
Analyzing the identity of LMP-1::GFP compartments in *glo-2(* −***)*** **embryos.** The majority of LMP-1::GFP containing compartments (marked with white arrows in all panels) lacked RAB-5 and RAB-7 in wild type (A–F) and *glo-2(zu455)* (J–O), however a subset of LMP-1::GFP colocalized with RAB-7 (black arrows). In both wild type (G–I) and *glo-2(zu455*) (P-R), the lysosomal hydrolase F11E6.1::mCherry localized to LMP-1::GFP containing organelles. In all panels, black arrowheads flank the intestine of 1.5-fold stage embryos.

While trafficking to, and the function of, lysosomes appeared normal in *glo-2(zu455)*, we found that this allele doubled the number of compartments containing the integral membrane protein LMP-1::GFP, which tended to be less concentrated near the apical surface of intestinal cells (Figure 2 Q–R). On average, there were 11±0.9 (mean±SEM) LMP-1::GFP organelles spread throughout the four cells that make up intestinal rings 2 and 3 in wild-type (n = 5 embryos) and 22±0.9 in *glo-2(zu455)* (n = 5 embryos) 1.5-fold stage embryos (p = <0.001; unpaired t-test). To determine whether the increased number of LMP-1::GFP containing compartments in *glo-2(zu455)* resulted from mislocalization of LMP-1::GFP, we compared the identity of LMP-1::GFP containing compartments in wild-type and *glo-2(zu455)* embryos. In wild type, LMP-1::GFP largely associated with conventional lysosomes, as 90% (n = 89 compartments from 5 embryos) of LMP-1::GFP compartments colocalized with the lysosomal hydrolase F11E6.1::mCherry (Figure 8 G–I). Similarly, in *glo-2(zu455)*, 89% (95 compartments from 5 embryos) of LMP-1::GFP compartments contained F11E6.1::mCherry (Figure 8 P–R). In both wild type (n = 100 compartments from 5 embryos) and *glo-2(zu455)* (n = 100 compartments from 5 embryos) only 10% of LMP-1::GFP compartments colocalized with the early endosome marker RAB-5 (Figure 8 A–C and J–L). In wild type, 30% (n = 122 compartments from 6 embryos) of LMP-1::GFP compartments contained the late endosome marker RAB-7 (Figure 8 D–F). Notably, only 15% (n = 200 compartments from 5 embryos) of LMP-1::GFP positive compartments contained RAB-7 (Figure 8 M–O) suggesting that *glo-2(zu455)* leads to a modest increase in the number of properly formed conventional lysosomes in embryonic intestinal cells, without altering the distribution of LMP-1::GFP relative to early and late endosomes. Other *glo* mutants display enlarged LMP-1::GFP containing compartments in later stage embryos [4], [36], [68], suggesting that alterations in conventional lysosome number/size is a common effect of defective gut granule biogenesis.

### Assessing AP-3 and GLO-2 Dependence of CDF-2::GFP, LMP-1, and PGP-2 Trafficking to Gut Granules

The AP-3 adaptor complex has an evolutionarily conserved role in trafficking to LROs and plays the most well understood role in LRO biogenesis [69]. Comparing the effects of *glo-2* and AP-3 deficiencies on sorting to gut granules can provide insights into the shared and unique functions of these factors.

**Figure 9:**
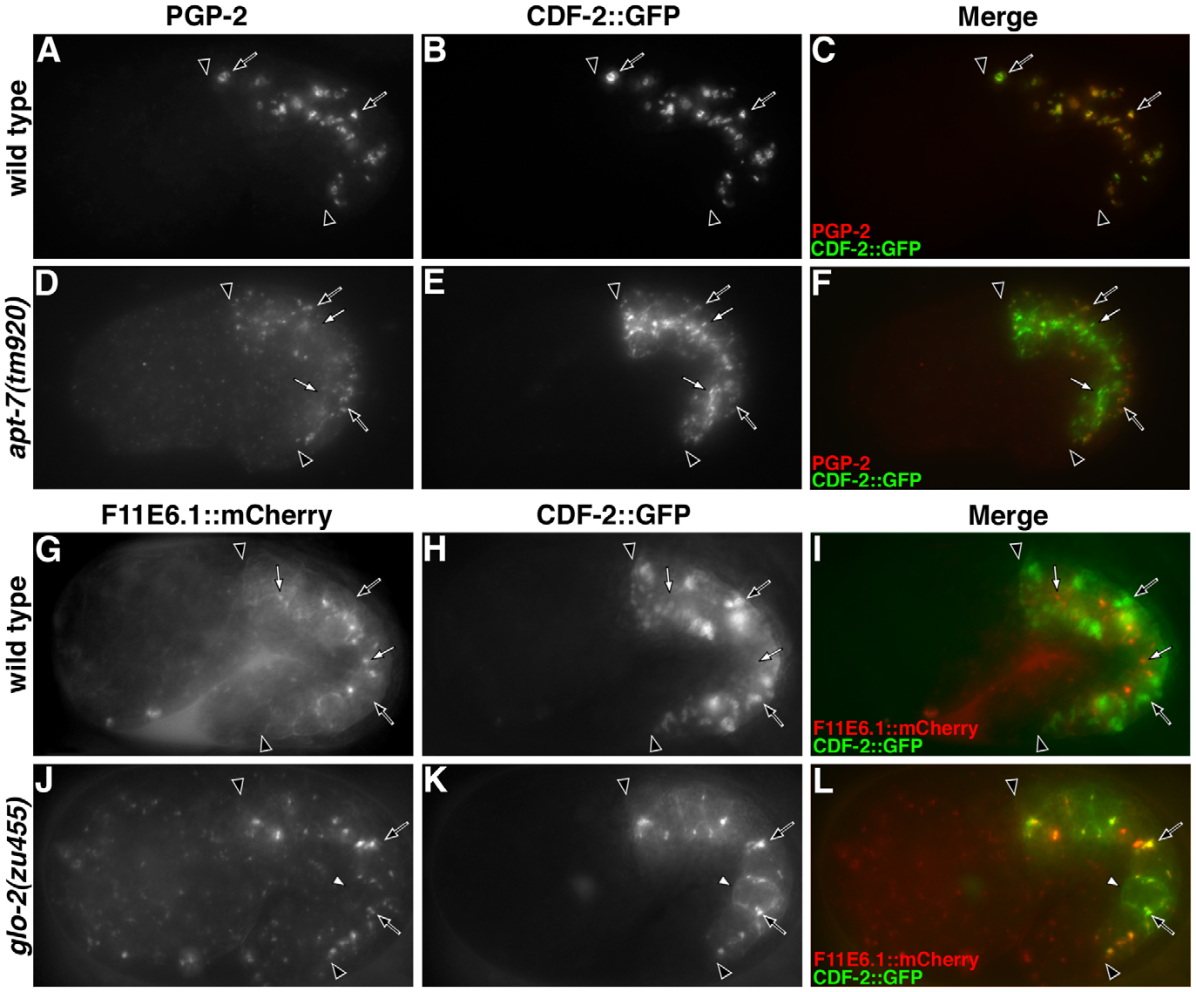
Analysis of CDF-2::GFP trafficking. In wild type, CDF-2::GFP (black arrows) colocalized with PGP-2 on gut granules (A–C) and CDF-2::GFP (black arrows) was not associated with F11E6.1::mCherry containing conventional lysosomes (white arrows) (G–I). (D–F) CDF-2::GFP was associated with PGP-2 containing gut granules (black arrows) and apical compartments (white arrows) in an AP-3 mutant. (J–L) PGP-2 was lacking and CDF-2::GFP was localized to conventional lysosomes (black arrows) and the plasma membrane (white arrowhead) in *glo-2(zu455)*. In all panels, black arrowheads flank the intestine of 1.5-fold stage embryos.

We investigated the gut granule directed trafficking of three integral membrane proteins, PGP-2, CDF-2::GFP and LMP-1 in *glo-2(*−*)* and AP-3(−) mutants. PGP-2 is a well-defined resident gut granule ABC transporter that appears to be exclusively localized to this organelle [36]. *cdf-2* encodes a zinc transporter homologous to mammalian ZnT2/ZnT3 [70]. A functional CDF-2::GFP fusion protein localized to gut granules marked with PGP-2 (Figure 9A–C) [33], [70]. Conventional lysosomes containing F11E6.1::mCherry were positioned near the apical surface of embryonic intestinal cells and lacked CDF-2::GFP (Figure 9 G–I). Thus, like PGP-2, CDF-2::GFP distribution is restricted to gut granules. LMP-1 is homologous to mammalian LAMP-1 and LMP-1::GFP is widely used as a lysosomal marker in *C. elegans* [71], [72]. In embryonic intestinal cells, LMP-1::GFP was not associated with PGP-2 marked gut granules (Figure 6A–C) and was enriched on the basolateral plasma membrane and largely associated with conventional lysosomes localized near the apical surface [33], [61]. However, endogenous LMP-1, detected with a newly developed and specific monoclonal antibody [35], was targeted to both gut granules and conventional lysosomes in embryos. Conventional lysosomes in *C. elegans* contain the integral membrane transporter CNTS-1 [73], [74]. Endogenous LMP-1 colocalized with CNTS-1::GFP (Figure 6D–F) and more basally positioned, PGP-2 containing gut granules (Figure 6G–H). In contrast to LMP-1::GFP, endogenous LMP-1 was not enriched on the plasma membrane of intestinal cells (Figure 6B, E, H).

The differences in the distribution of LMP-1::GFP and endogenous LMP-1 possibly result from the addition of GFP to the carboxy terminus of LMP-1 leading to disruption of its tyrosine-based sorting sequence [71], which is known to be recognized by the AP-3 adaptor complex [75]. If this is the case, loss of AP-3 activity should lead to a redistribution of LMP-1 so that it resembles that of LMP-1::GFP. Mutations disrupting the function of *C. elegans* AP-3 subunits APT-6 and APT-7 result in a reduction, but not a complete loss of embryonic gut granules [4], [36]. We found that PGP-2 containing gut granules in *apt-6(*−*)* embryos lacked LMP-1 (Figure 6J–L), indicating that AP-3 activity was essential for LMP-1 trafficking to gut granules. Based upon the apical distribution of LMP-1 containing organelles in *apt-6(*−*)* gut cells, endogenous LMP-1 appeared to be trafficked to conventional lysosomes in AP-3(−) embryos (Figure 6K). Thus, LMP-1 distribution in AP-3(−) mirrors that of LMP-1::GFP in wild type. The sorting of LMP-1::GFP to conventional lysosomes was not disrupted by *apt-7(*−*)* (data not shown). These results show that LMP-1::GFP trafficking differs from LMP-1 and suggests that LMP-1::GFP is mistargeted to the plasma membrane where it is endocytosed and subsequently transported to conventional lysosomes via an AP-3 independent pathway. Moreover, these data indicate that LMP-1 sorting to gut granules requires AP-3 function, while PGP-2 does not.

The proper trafficking of mammalian ZnT3, the homolog of CDF-2, requires AP-3 [76]. The AP-3 adaptor complex directly interacts with ZnT3 through a cytoplasmic [D/E]XXXL[L/I] targeting sequence, which is conserved in CDF-2 [77]. In *apt-7(*−*)* embryos we found that CDF-2::GFP was still localized, albeit at decreased levels, to PGP-2 containing gut granules and that a substantial proportion of CDF-2::GFP was mislocalized to what are likely apical conventional lysosomes (Figure 9D–F). Thus, AP-3 is not essential for the sorting of CDF-2::GFP to gut granules, instead it prevents the trafficking of CDF-2::GFP to conventional lysosomes.

Our results demonstrate that LMP-1 trafficking to gut granules is AP-3 dependent and that CDF-2::GFP and PGP-2 trafficking to gut granules is partially independent of AP-3 activity. Unlike AP-3(−) embryos that have PGP-2 containing organelles, *glo-2(zu455)* lacked distinct PGP-2 marked compartments (Figure 6M). In *glo-2(zu455)*, LMP-1 was found associated with apical organelles and was lacking from the region of intestinal cells where it associates with gut granules in wild type (Figure 6N). Unlike wild type, where CDF-2::GFP was exclusively localized to gut granules (Figure 9A–C), in *glo-2(zu455)* embryos the reporter was enriched on the plasma membrane and apical F11E6.1::mCherry containing conventional lysosomes (Figure 9J–L). Therefore, *glo-2(+)* activity is essential for the proper sorting of all three of these proteins to gut granules.

### *snpn-1(*−*)* Exhibits Defective Trafficking to Gut Granules

*snpn-1(tm1892)* animals were viable and displayed a complete loss of birefringent and autofluorescent gut granules (Figure 5 C–F), suggesting that they were defective in gut granule biogenesis. Consistent with this idea, we found that *snpn-1(tm1892)* embryos lacked PGP-2 containing organelles, displayed dramatically altered morphology of GFP::GLO-1, and CDF-2::GFP was mislocalized to the plasma membrane and apical organelles, which are likely conventional lysosomes (Figure 5G–L). *snpn-1(*−*)* did not alter the morphology and subcellular distribution of early endosomes containing RAB-5 or late endosomes containing RAB-7 (Figure 5M–P). Moreover, yolk platelets marked with YP170::GFP were similar in *snpn1(*−*)* and wild-type embryos (Figure 5 Q–R), suggesting that the trafficking to and degradative properties of conventional lysosomes were not altered by loss *of snpn-1(*−*)* activity. The phenotypes exhibited by *snpn-1(*−*)* are similar to *glo-2(*−*)*, suggesting that they function together, likely as part of the BLOC-1 complex, to promote trafficking to gut granules.

### *glo-2(*−*)* and *snpn-1(*−*)* are Epistatic to AP-3(−)

Biochemical studies show that AP-3 and BLOC-1 can reside on the same membranes and can physically interact [11], [13], suggesting a tight functional linkage [78]. However, our studies of PGP-2 and CDF-2::GFP trafficking, suggest that AP-3 and BLOC-1 are differentially required in the sorting of these proteins to gut granules. Reduced numbers, but not a complete loss, of birefringent and autofluorescent gut granules in embryos and adult intestinal cells, respectively, were seen in *apt-6(ok429)* and *apt-7(tm920)* animals, which likely lack all AP-3 activity (Tables 1 and 2). Thus, genes required for the formation of these gut granules must have activities promoting gut granule biogenesis that are independent of AP-3 function and in genetic tests would be epistatic to AP-3(−). Significantly, gut granules were completely absent in *glo-2(zu455)*; *apt-7(tm920)*, *snpn-1(tm1892)*; *apt-6(ok429)*, and *snpn-1(tm1892)*; *apt-7(tm920)* double mutants (Tables 1 and 2), indicating that *glo-2(*−*)* and *snpn-1(*−*)* are epistatic to AP-3(−) and that *glo-2(+)* and *snpn-1(+)* mediate the formation of gut granules in animals lacking AP-3 activity. Thus, these genes, and by extension the BLOC-1 complex, must have some functions that act independently of AP-3 in gut granule biogenesis. However, these results do not preclude the prospect that BLOC-1 could also function with AP-3 in the same trafficking pathway, possibly even via direct physical interactions.

## Discussion

Our work shows that BLOC-1 function in LRO biogenesis is conserved in *C. elegans*. Mutations disrupting two BLOC-1 encoding subunits *glo-2* and *snpn-1* similarly alter the biogenesis gut granules. We demonstrate that BLOC-1 subunits in *C. elegans* (1) show physical interactions similar to mammalian and *Drosophila* homologues, (2) are required for the proper trafficking of gut granule cargo, and (3) as seen in other systems, appear to function partially independent of AP-3 activity.

### Genetics of *C. elegans* BLOC-1 Alleles

The *snpn-1(−)* allele and two *glo-2(−)* alleles we used in our studies have distinct effects on gut granule biogenesis. Based upon numbers of embryonic and adult gut granules, the relative mutant strengths are *snpn-1(tm1892)* > *glo-2(zu455)* > *glo-2(tm592)* (Tables 1 and 2). *snpn-1(tm1892)* exhibited the strongest Glo phenotype, completely lacking embryonic and adult gut granules. *glo-2(zu455)* lacked embryonic gut granules, however some gut granules were present in adults (Tables 1 and 2, Figures 2 and 7), which could result from partial activity of *glo-2(zu455)* or compensating activity being present in larvae/adults. *glo-2(tm592)* was cold sensitive for the embryonic Glo phenotype and exhibited more adult autofluorescent gut granules than *glo-2(zu455)* (Tables 1 and 2). Placing *glo-2(tm592)*, but not *glo-2(zu455)*, over a chromosomal deficiency removing *glo-2* resulting in an enhanced Glo phenotype, indicating that *tm592* retained partial *glo-2(+)* function (Table 2). The molecular lesions affecting these genes are consistent with the graded phenotypic effects. The *snpn-1(tm1892)* deletion removes the upstream regulatory sequences, the translation initiating ATG, and the first exon of *snpn-1* and therefore likely is null (Figure 5A). *glo-2(zu455)* is a splice acceptor mutation in the 5′ end of exon 4, which leads to the production of *glo-2.a* transcripts with altered 3′ sequences and is not predicted to alter *glo-2.b* (Figure 1A and methods). While *glo-2(zu455)* is not obviously a molecular null allele, it nevertheless severely compromises *glo-2(+)* activity. The *glo-2(tm592)* deletion removes part of the 5′ regulatory region without altering the *glo-2* coding sequences (Figure 1A) and clearly retains partial activity (Figure 3). While it is possible that the different Glo phenotypes exhibited by *glo-2* and *snpn-1* mutants result from GLO-2 and SNPN-1 having independent functions in gut granule biogenesis, their homology with mammalian BLOC-1 subunits, their conserved physical interactions, and their similar trafficking phenotypes strongly suggest that the *snpn-1* and *glo-2* alleles differentially compromise BLOC-1 activity.

We found that *glo-2* encodes two different transcripts, which are both predicted to encode short proteins composed almost exclusively of alpha-helical heptad repeats (Figure 1A). The heptad repeat containing domain of mammalian Pallidin mediates association with itself, BLOS1, and BLOS4 [46], [51], [79]. Our 2-hybrid studies with *C. elegans* proteins show that GLO-2 also interacts with itself, BLOS-1, and BLOS-4 (Figure 1D). *glo-2.a* and *glo-2.b* only differ by the inclusion of exon 4 (Figure 1A), which is not predicted to code for heptad repeats. Sequences encoded by this exon are not conserved in distant taxa (not shown) or closely related nematodes (Figure 1B), suggesting that it might not be significant in GLO-2 activity. In support of this idea, expression of *glo-2.b*, which lacks this exon restored gut granule formation in *glo-2(zu455)*. Possibly the adult granules in *glo-2(zu455)* result from the activity of *glo-2.b*. We think it likely that both *glo-2.a* and *glo-2.b* contribute to gut granule biogenesis and that altered splicing in *glo-2(zu455)* results in the addition of 3′sequences, which disrupt the function and/or stability of *glo-2.a*, reducing GLO-2 activity to a level insufficient for proper gut granule biogenesis.

The *in vitro* evidence for Pallidin being a component of BLOC-1 is quite strong [79]. However, at present the evidence for Pallidin acting to mediate LRO biogenesis as part of BLOC-1 *in vivo* is limited to the phenotypic similarities of mice mutant for different BLOC-1 subunits. Our genetic studies with *glo-2(−)* alleles support GLO-2 functioning as part of a multi-protein complex, likely BLOC-1, in gut granule formation. Placing the weak *glo-2(tm592)* allele over the strong loss of function *glo-2(zu455)* allele resulted in a stronger Glo phenotype than when *glo-2(tm592)* was placed over *hDf6*, a deficiency removing *glo-2(+)* (Table 2). Based upon the strength of the alleles, we expected *glo-2(tm592)*/*glo-2(zu455)* to exhibit a similar, or possibly weaker Glo phenotype, than *glo-2(tm592)*/*hDf6*. While it is possible that the removal of genes by *hDf6* ameliorate the Glo phenotype of *glo-2(tm592)*, based upon our studies showing that *glo-2(zu455)* encodes mutant GLO-2 proteins that retain the heptad repeats but have altered C-termini, we favor the interpretation that *glo-2(zu455)* dominantly interferes with *glo-2(+)* function encoded by *glo-2(tm592)*. *glo-2(tm592)* alters *glo-2* regulatory sequences, possibly diminishing GLO-2 levels. Thus, if GLO-2 interacts with itself or other proteins to promote gut granule biogenesis, then reduced levels of GLO-2 in *glo-2(tm592)* could be sensitive to competition with non- or partially functional forms of GLO-2 encoded by *glo-2(zu455)*. We believe that our RNAi results point to BLOS-1 and BLOS-4 being candidate GLO-2 interacting proteins *in vivo*. *glo-2(tm592)* was much more sensitive to RNAi targeting *blos-1* and *blos-4* than other BLOC-1 subunit genes (Figure 3). Possibly, a subcomplex containing GLO-2, BLOS-1, and BLOS-4, which has been seen *in vitro* with mammalian homologues [79], becomes limiting for gut granule biogenesis in *glo-2(tm592)*, making its subunits more susceptible to depletion by RNAi.

### BLOC-1 and AP-3 Function in *C. elegans*

Our work provides new insights into BLOC-1 subunit composition and function in metazoan LRO formation. Prior to this study, mutations in only 6 of the 8 currently recognized BLOC-1 subunits were known to cause defects in LRO biogenesis [10], [17]. We show for the first time in any system that Snapin is required for LRO biogenesis (Tables 1, 2 and Figure 5). Moreover, we find that *blos-2* is required for proper gut granule formation (Figure 3E), which together with studies of *blos2(−)* in the formation of silkworm urate granules [80], provide the first genetic evidence that BLOS2 functions in LRO biogenesis. Recently, a BLOC-1 interacting protein called KXD1 has been shown to function in LRO biogenesis in mice [81]. KXD1 is homologous to C13F10.2 [16], which we did not implicate in gut granule formation (Figure 3E). However, KXD1 contains a KxDL motif that is present in Y37E11B.2 [16], which we show likely functions in gut granule formation (Figure 3E). Thus, KxDL containing proteins represent strong candidates as new BLOC-1 subunits.

The role of BLOC-1 in the biogenesis of LROs has only been carefully examined in mammalian melanocytes. In this cell type, BLOC-1(*−*) mutants generate lysosome-related melanosomes, however they lack pigment due to defects in trafficking a subset of melanosomal proteins [8], [14], [82]. In contrast to what has been seen for LROs in melanocytes, our results strongly suggest that disrupting BLOC-1 activity abolishes LRO biogenesis in *C. elegans* intestinal cells. In *glo-2(zu455)* embryos, three different gut granule associated membrane proteins were lacking from intestinal cells or completely mislocalized to other organelles (Figures 2, 6, 9) and two different vital dyes that mark gut granules as well as birefringent gut granule material did not accumulate in intestinal cells (Figure 2). Similar effects were seen in *snpn-1(tm1892)* embryos (Figure 5). Possibly, the more pronounced defects in LRO formation result from *C. elegans* BLOC-1 mediating the trafficking of factors that facilitate gut granule biogenesis. For example, PGP-2 promotes gut granule biogenesis [36] and it is lacking in *glo-2(−)* and *snpn-1(−)* intestinal cells (Figures 5 and 6), possibly due to defects in sorting (discussed below). Alternatively, BLOC-1 independent trafficking pathways not present in *C. elegans* intestinal cells might promote LRO biogenesis in mammalian melanocytes.

Our work highlight similar as well as different effects of BLOC-1(*−*) on protein localization and trafficking to *C. elegans* gut granules and mammalian melanosomes. Proteins whose trafficking to melanosomes is disrupted by BLOC-1(−) accumulate at the plasma membrane, early endosomes, and can be mistargeted to conventional lysosomes where they are degraded, leading to a decrease in their steady state levels [8], [11], [14], [82]. CDF-2::GFP and PGP-2 are transmembrane proteins that in wild-type embryos are only detected at gut granules (Figure 6 and 9). Similar to studies of melanosomal proteins, in BLOC-1(−) embryos, CDF-2::GFP was found at the plasma membrane and was enriched at conventional lysosomes (Figures 5 and 9). We saw no evidence that CDF-2::GFP accumulated at early endosomes. PGP-2 could not be detected by immunofluorescence microscopy in BLOC-1(−) embryos (Figures 5 and 6), a phenotype that has not been reported for any melanosomal protein in BLOC-1(−). The loss of PGP-2 might result from PGP-2 and other gut granule proteins being trafficking to conventional degradative lysosomes, leading to the increased number of LMP-1::GFP containing conventional lysosomes that we see in *glo-2(*−*)* (Figure 2). LMP-1 is localized to wild-type gut granules, which are located basolaterally, and conventional lysosomes, which are positioned near the apical membrane of embryonic intestinal cells (Figure 6). In *glo-2(*−*)* embryos, LMP-1 still localized to conventional lysosomes, however the protein was lacking from organelles resembling gut granules (Figure 6). Notably, trafficking of the orthologous protein LAMP-1 to melanosomes is not disrupted by BLOC-1(−) [14]. At present, we cannot determine whether the dissimilar effects on gut granule and melanosomal protein sorting reflect differences in BLOC-1 function and/or cell type specific variations in LRO biogenesis pathways. In either case, our results affirm the importance of analyzing the function of sorting complexes in multiple cell types and experimental systems.

To investigate the differences between sorting to gut granules and melanosomes, we analyzed the localization of gut granule marker proteins in cells lacking AP-3 function. AP-3 is a conserved heterotetrameric complex that facilitates LRO biogenesis in mammals, *D. melanogaster*, and *C. elegans* [69]. Mammalian melanosomes are generated in AP-3(−) melanocytes [14], [82], [83], however they lack a subset of melanosomal proteins, which are distinct from those lacking in BLOC-1(−) [83], [84]. Similarly, gut granules with altered protein composition are generated in *C. elegans* AP-3(−) mutants. In prior studies, we have shown that AP-3(−) embryos retain gut granules, however they mislocalize birefringent material extracellularly into the intestinal lumen [4]. Here we demonstrate that gut granules in AP-3(−) embryos contained PGP-2 and CDF-2::GFP (Figure 9). However, loss of AP-3 activity caused a substantial fraction of CDF-2::GFP to likely become missorted to the plasma membrane and conventional lysosomes (Figure 9). Similarly, mammalian AP-3 is required for the proper sorting of the CDF-2 homologue ZnT3 to synaptic vesicles in neuronal cells [12], [85]. AP-3 has a well characterized role in sorting LAMP-1 in mammalian cells [86], [87], [88], however the role of AP-3 in trafficking LAMP-1 toward LROs, where it is known to reside [89], is currently unclear. In *C. elegans* gut cells, we find that AP-3 activity is necessary for the sorting of LMP-1 to gut granules, however LMP-1 is still trafficked to conventional lysosomes (Figure 6). In general, the effects of AP-3(−) on gut granule biogenesis closely mirror what has been seen for melanosomes.

Despite well-documented physical interactions between BLOC-1 and AP-3 [11], [90], genetic studies, including ours with *C. elegans*, support the view that BLOC-1 function is not limited to the activity of AP-3. First, in *C. elegans* (Figures 6 and 9), mice [91], and *D. melanogaster* [17], BLOC-1(−) and AP-3(−) single mutants have different phenotypic effects on LRO biogenesis. Second, in mice and *D. melanogaster*, BLOC-1(−); AP-3(−) double mutants exhibit stronger defects in LRO formation than the single mutants [17], [92]. Third, we find that *C. elegans* BLOC-1 is required for the biogenesis of gut granules in mutants that likely lack all AP-3 activity (Tables 1 and 2). Fourth, similar functional relationships between AP-3 and BLOC-1 have been seen in cells that lack LROs [13]. These conserved genetic interactions likely reflect a fundamental and currently poorly understood relationship between BLOC-1 and AP-3 function in membrane sorting.

Many organisms, including *C. elegans* [58], [59], express BLOC-1 subunits in cell types that lack LROs [45], [50], [93]. Thus, it is likely BLOC-1 promotes the sorting of proteins in cells without LROs that play important physiological roles in *C. elegans*. For example, BLOC-1 has been implicated in neuronal function and disease [78], thus the *C. elegans* nervous system is a likely site of activity for this complex. However, the effects of BLOC-1(−) in cells outside the intestine are likely to be subtle as BLOC-1(−) intestinal cells did not show any obvious alterations of organelles in the conventional endolysosomal pathway that are indicative of sorting defects (Figures 2 and 5) and severely disrupting endolysosomal trafficking in *C. elegans* leads to pronounced phenotypes that are not displayed by *glo-2(*−*)* or *snpn-1(*−*)* [65], [72].

## Supporting Information

Figure S1 **Alignment of** ***C. elegans*** **(Ce) BLOC-1 subunits to (Dm)** ***D. melanogaster*** **and (Hs) human homologues.** The sequences under the lines denote highly conserved motifs identified by Cheli & Dell’Angelica (2010). The following sequences were used for (A) BLOS1: human NP_001478, *D. melanogaster* NP_725401, *C. elegans* NP_499262; (B) BLOS2: human NP_776170, *D. melanogaster* NP_648427, *C. elegans* NP_500967; (C) BLOS4: human NP_060836, *D. melanogaster* NP_648414, *C. elegans* NP_495247; (D) Dysbindin: human NP_115498, *D. melanogaster* NP_649064, *C. elegans* NP_492628; (E) Muted: human NP_958437, *D. melanogaster* NP_001036744, *C. elegans* NP_501119.
(PDF)
