# Supplementary material for: C. elegans BLOC-1 Functions in Trafficking to Lysosome-Related Gut Granules
Source: PLoS One. 2012 Aug 15;7(8):e43043. doi: 10.1371/journal.pone.0043043 (PMC3419718; doi:10.1371/journal.pone.0043043)
Supplement: Figure S1 — Alignment of C. elegans (Ce) BLOC-1 subunits to (Dm) D. melanogaster and (Hs) human homologues. The sequences under the lines denote highly conserved motifs identified by Cheli & Dell’Angelica (2010). The following sequences were used for (A) BLOS1: human NP_001478, D. melanogaster NP_725401, C. elegans NP_499262; (B) BLOS2: human NP_776170, D. melanogaster NP_648427, C. elegans NP_500967; (C) BLOS4: human NP_060836, D. melanogaster NP_648414, C. elegans NP_495247; (D) Dysbindin: human NP_115498, D. melanogaster NP_649064, C. elegans NP_492628; (E) Muted: human NP_958437, D. melanogaster NP_001036744, C. elegans NP_501119. (PDF) [file pone.0043043.s001.pdf]

Supplemental Figure 1
Hermann et al.

A

```
CeBLOS-1  ----MLKEHSKKQHLRREVQEKLKNEAIVAAQTLSTAVVDHLNAKVAQAYGNQKRLDVEA 56
DmBLOS1   MLTSMVKEHHKEQAKRKQEQEVRRKEAIEASNELTQSLVDTLNVGVAQAYLNQKRLDAEA 60
HsBLOS1   MLSRLLKEHQAKQNERKELQEKRRREAITAATCLTEALVDHLNVGVAQAYMNQRKLDHEV 60

CeBLOS-1  KRFENNSAALAKQTEQWLFITEGLNYALKEIGDVENWSKTIENDMKIITETLRRAYEAKN 116
DmBLOS1   KQLHLGATNFAKQTHQWLQLIDQFSTALKDLGDVENWARSIEGDMHTINQTLELAYKASR 120
HsBLOS1   KTLQVQAAQFAKQTGQWIGMVENFNQALKEIGDVENWARSIELDMRTIATALEYVYKGQL 120

CeBLOS-1  PPLPPN--------------QANPASH 129
DmBLOS1   ATQTSSGAGTSLEASTSASASANPSAT 147
HsBLOS1   QSAPS---------------------- 125
```

B

```
CeBLOS-2  -------MAEINERASTSSPPVPST-------------PAPVPHIRQLADNMTDKVGQFF 40
DmBLOS2   MDKPTTSAAAAAAQDSNLLPDSPQHGPTLSSASSFEALTRHDPNLSRLATKMFNKTEEYI 60
HsBLOS2   -------MAAAAEGVLATRSDEPAR-DDAAVETAEEAKEPAEADITELCRDMFSKMATYL 52

CeBLOS-2  QHQLEGSIEEYKLLETMNNTTAQRYVDMKVVAEKVAGKLDNLNQKYENLRPYLSQIDAMD 100
DmBLOS2   THELNAPLEDYKLLEEMNKATIAKYKDMRQIAENLNTSTSELSLKFQQLAPMMQQIDEIS 120
HsBLOS2   TGELTATSEDYKLLENMNKLTSLKYLEMKDIAINISRNLKDLNQKYAGLQPYLDQINVIE 112

CeBLOS-2  ESTRRLEEATAVLENYVTQLESKLTNIQQQSQ------- 132
DmBLOS2   DTVDKLEAAAYKLDAYSIALENRVKCVLQRKSGGGQVAQ 159
HsBLOS2   EQVAALEQAAYKLDAYSKKLEAKYKKLEKR--------- 142
```

C

```
CeBLOS-4      -------------------------------------------------------MTGVD 5
DmBLOS4       ---------------------------------------------------------MQS 3
HsCappuccino  MEGSFSDGGALPEGLAEEAEPQGAAWSGDSGTVSQSHSSASGPWEDEGAEDGAPGRDLPL 60

CeBLOS-4      DISSNVHKLVGVTAGMNTAASTGPMNLVLDKCSVVLEELR-TIQILTETHSEGLSEQLKM 64
DmBLOS4       NIENVSRDYAKILQSADLEKEINPLCTNIEDMLARLDEFETLLASVRAESNGMMANNVCS 63
HsCappuccino  HRRAAAGYAACLLPGAGARPEVEALDASLEDLLTRVDEFVGMLDMLRGDSSHVVSEGVPR 120

CeBLOS-4      TEKNILEMENLFDQIDQLCLFVQKAKSDLDKLEKLYNVVDRQ------------------ 106
DmBLOS4       ILGFTDSFEQLKARIDGLEQCVGVVSANLSEVERSVDIAEEELHVTDYSLKGLLLKPLKA 123
HsCappuccino  IHAKAAEMRRIYSRIDRLEAFVRMVGGRVARMEEQVTKAEAELGTFPRAFKKLLHT---- 176

DmBLOS4       KLSASDTSTLSSLPRSNLVEEEYQPVEIYKSDDYFGKSEEENYVAK 169
HsCappuccino  ---MNVPSLFSKSAPSRPQQAGYEAPVLFRTEDYFPCCSERPQL-- 217
```

D

```
CeDSBN-1      MDMLNTLRDTLSSVQAE------------------------LSTGVEK------LRMNVS 30
DmDysbindin   --MFGNLKKKLSSAIQEGLVISENLQQQYRQRVSSGNSGSSQASGITTPISPLGLNESLS 58
HsDysbindin   --MLETLRERLLSVQQD------------------------FTSGLKT------LSDKSR 28

CeDSBN-1      ANIVAQQKVSTESVN-----EILNTSAGNELLQNFQNLITEVEENGAEGGRLANLCSTRM 85
DmDysbindin   SSRSSSLSLSAPFQLTDGVPSHLNVAAGCSLLAKYEDDWQQIHGANEKNAEKAAQIANQI 118
HsDysbindin   EAKVKSKPRTVPFLP--------KYSAGLELLSRYEDTWAALHRRAKDCASAGELVDSEV 80

CeDSBN-1      GRCQQMCKEKADAVMEIDEFLRNSVEFDKKIREINAQISKLTRFCNQTEQAMTYLEALCE 145
DmDysbindin   SGIQDQASHQHRIMSELNSSLAGIPTLIAQLQNSSQVLNSLEEMGKQLEIELEKLEDLRE 178
HsDysbindin   VMLSAHWEKKKTSLVELQEQLQQLPALIADLESMTANLTHLEASFEEVENNLLHLEDLCG 140

CeDSBN-1      VAHTEG-----EVDLIRQQAKSAATIVQIECESPSVLTSALRSRPEDAVKAQQE------ 194
DmDysbindin   ECELQEFILEQQFQLSRHKQKKLNELEQYRQQIAQKHQSKIKDQEQTLLKLQRERQAVFD 238
HsDysbindin   QCELERCKHMQSQQLENYKKNKRKELETFKAELDAEHAQKVLEMEHTQQMKLKERQKFFE 200

CeDSBN-1      ---EVMLEEFLSSKNMQLP----------------------------------------- 210
DmDysbindin   DAFREDMEEYKQRGQLTKIQTTSNKLALEEVVLEAN--------EVETKDALEQFLNG-- 288
HsDysbindin   EAFQQDMEQYLSTGYLQIAERREPIGSMSSMEVNVDMLEQMDLMDISDQEALDVFLNSGG 260

HsDysbindin   EENTVLSPALGPESSTCQNEITLQVPNPSELRAKPPSSSSTCTDSATRDISEGGESPVVQ 320

HsDysbindin   SDEEEVQVDTALATSHTDREATPDGGEDSDS 351
```

E

```
CeMUTD-1  ----------------------------MATIPSVVREITLVGEQIFDHTQVVRAEIDRF 32
DmMuted   ---------------------------MKIMISQVGRELYKVPLRILDHRVFVNGEIEAF 33
HsMuted   MSGGGTETPVGCEAAPGGGSKKRDSLGTAGSAHLIIKDLGEIHSRLLDHRPVIQGETRYF 60

CeMUTD-1  VERFERNERHREFDGILRASHAL-----------VESSETPVEGLFDMG-KMEHMTQCVD 80
DmMuted   LENFEVRRNDSEVEKLFQVTETVGSLKYDLSRCSATGRGSGAENLAQLDTEVSHLLDGVN 93
HsMuted   VKEFEEKRGLREMRVLENLKNMIHETN---EHTLPKCRDTMRDSLSQVLQRLQAANDSVC 117

CeMUTD-1  DITKKLQT--------LVEPKYQKEHD--VYLEKVKEDQKKYVDVCREQAMNKMRSMTAH 130
DmMuted   ALLAKAKVER-TASTQLQEARLAREQRRAEFLTNLEHGYRRIENSFEEKEEEIAELYSDL 152
HsMuted   RLQQREQERKKIHSDHLVASEKQHMLQWDNFMKEQPNKRAEVDEEHRKAMERLKEQYAEM 177

CeMUTD-1  R--------- 131
DmMuted   QLKLNIAK-- 160
HsMuted   EKDLAKFSTF 187
```
